# Machine Learning Identification of Cell-Type-Specific Molecular Signatures Distinguishing COVID-19 from Other Lower Respiratory Tract Diseases

**DOI:** 10.3390/life16050771

**Published:** 2026-05-04

**Authors:** Yusheng Bao, Xianchao Zhou, Lei Chen, Kaiyan Feng, Wei Guo, Tao Huang, Yu-Dong Cai

**Affiliations:** 1 School of Life Sciences, Shanghai University, Shanghai 200444, China; bys@shu.edu.cn; 2 Center for Single-Cell Omics, School of Public Health, Shanghai Jiao Tong University School of Medicine, Shanghai 200025, China; zhouxianchao@sjtu.edu.cn; 3 College of Information Engineering, Shanghai Maritime University, Shanghai 201306, China; lchen@shmtu.edu.cn; 4 Department of Computer Science, Guangdong AIB Polytechnic College, Guangzhou 510507, China; kyfeng@gdaib.edu.cn; 5 Shenzhen Institute of Advanced Technology, Chinese Academy of Sciences, Shenzhen 518055, China; gw_1992@sjtu.edu.cn; 6 Bio-Med Big Data Center, CAS Key Laboratory of Computational Biology, Shanghai Institute of Nutrition and Health, University of Chinese Academy of Sciences, Chinese Academy of Sciences, Shanghai 200031, China; 7 Department of Artificial Intelligence and Digital Health, CAS Engineering Laboratory for Nutrition, Shanghai Institute of Nutrition and Health, University of Chinese Academy of Sciences, Chinese Academy of Sciences, Shanghai 200031, China

**Keywords:** COVID-19, lower respiratory tract diseases, single cell transcriptomics, machine learning, population-specific immunity, African genomics

## Abstract

Coronavirus Disease 2019 (COVID-19) and other lower respiratory tract diseases (LRTDs), including bacterial pneumonia and acute respiratory distress syndrome, share overlapping clinical features but arise from distinct pathophysiological mechanisms. The molecular signatures that distinguish these diseases remain insufficiently characterized in African populations, where genetic background, endemic infections, and environmental exposures may substantially shape immune responses. We integrated spatially resolved single-cell transcriptomic profiles from lung autopsy specimens of 30 Malawian patients, including 10 with COVID-19, 12 with other LRTDs, and 8 non-LRTD controls. In total, 61,391 cells representing 15 cell types and 36,602 gene expression features were analyzed. Using an integrated machine learning framework that combined nine feature-ranking algorithms with incremental feature selection, we identified potential molecular signatures that could discriminate among disease states within this cohort. The optimal classification models achieved weighted F1 scores greater than 0.94, demonstrating a robust capacity to differentiate COVID-19 from other LRTDs in our dataset. Notably, the macrophage-associated state in COVID-19 was dominated by an IFN-γ response with upregulation of CD163 and HLA-DQA2, contrasting sharply with the type I/III interferon signature reported in European cohorts. In addition, we observed cell-type-specific COVID-19 signatures, including downregulation of CAV1 in AT1 cells, consistent with epithelial damage; dysregulation of SFTPC in AT2 cells, suggesting surfactant dysfunction; and upregulation of NFKBIA in neutrophils, indicating altered inflammatory regulation. Gene Ontology enrichment further revealed universal disruption of protein synthesis machinery, along with cell-type-specific alterations in immune activation, epithelial repair, and inflammatory signaling pathways.

## 1. Introduction

Coronavirus Disease 2019 (COVID-19), along with other lower respiratory tract diseases (LRTDs) such as severe pneumonia and acute respiratory distress syndrome (ARDS), remains a major cause of morbidity and mortality worldwide. LRTDs, including community-acquired pneumonia, bacterial pneumonia, and ARDS, are common pulmonary conditions with high fatality rates [1]. Although these diseases share certain clinical manifestations and pathological features, including diffuse alveolar damage, inflammatory responses, and impaired respiratory function, their distinct etiologies lead to different disease courses and patterns of tissue injury. COVID-19, caused by severe acute respiratory syndrome coronavirus 2 (SARS-CoV-2), overlaps clinically with other forms of pneumonia but also exhibits pathophysiological features that have drawn considerable attention [1]. Studies have shown that patients with COVID-19 often develop severe endothelial injury, widespread microthrombi, and pulmonary angiogenesis, changes that extend beyond those typically observed in conventional pneumonia and suggest distinct mechanisms at the level of the pulmonary microcirculation. In addition, the immunopathology of COVID-19 is highly atypical, characterized by a dysregulated state that combines immune hyperactivation with immunosuppression, in contrast to the neutrophil-dominant inflammatory responses commonly seen in traditional bacterial or viral infections [2].

A better understanding of the cellular and molecular processes underlying these life-threatening pulmonary complications is essential for the development of effective therapeutic strategies. Single-cell transcriptomic analysis has advanced rapidly and has emerged as a powerful tool for exploring lung disease pathobiology at unprecedented cellular resolution. Several single-cell studies of COVID-19 have reported major changes in the frequency and composition of pulmonary immune cells, including abnormal accumulation of I-M2-like monocytic-macrophage cells, loss of alveolar macrophage function, exhaustion of CD4+/CD8+ T cells, E. coli-like epithelial cell senescence, failure of regeneration, and activation of procoagulative transcriptional modules [3,4,5]. These alterations may be closely linked to pulmonary complications driven by direct viral cytopathic effects as well as virus-triggered systemic hyperinflammation. In contrast, single-cell analyses of bacterial pneumonia have highlighted a central role for neutrophils and M1-like classically activated cells in acute inflammation, while adaptive immunity appears relatively preserved [6].

Nevertheless, prior studies have focused largely on participants from Europe and Asia, with limited representation from Africa. As a result, our understanding of disease pathogenesis across geographically diverse populations remains incomplete [7,8]. In African settings, and particularly in low-income countries such as Malawi, common coinfections and distinct environmental exposures may alter host immune responses to viral infection and thereby influence disease course and therapeutic targets. Notably, the original study of the Malawian cohort reported that, although fatal COVID-19 shared certain histopathological features with cases from other regions, its immune signature differed from patterns described in some non-African cohorts. In the Malawian cohort, IFN-γ-associated responses were prominent in lung-resident macrophages, whereas type I/III interferon-related responses were reported in blood-derived monocytes from US, European, and Asian cohorts [9]. Such population-specific immunological signatures point to divergent pathogenic mechanisms in the same disease and underscore the importance of conducting single-cell studies in underrepresented and diverse populations.

To address these gaps, the present study integrates single-cell transcriptomic data with machine learning (ML) methods. Specifically, we developed an ML-based analytical framework that includes nine feature-ranking algorithms, incremental feature selection (IFS) [10], two classification algorithms, and the Synthetic Minority Oversampling Technique (SMOTE) [11]. We applied this framework to investigate gene expression features across COVID-19, LRTDs, and non-LRTD controls. We focused on the overlap among salient features identified by multiple ranking algorithms to isolate the most robust and disease-specific signatures. Our aim was to characterize the cellular and immune microenvironmental features associated with COVID-19 relative to other LRTDs and to provide a transcriptomic framework for hypothesis generation in diverse populations.

## 2. Materials and Methods

### 2.1. Dataset Description

**Figure 1:**
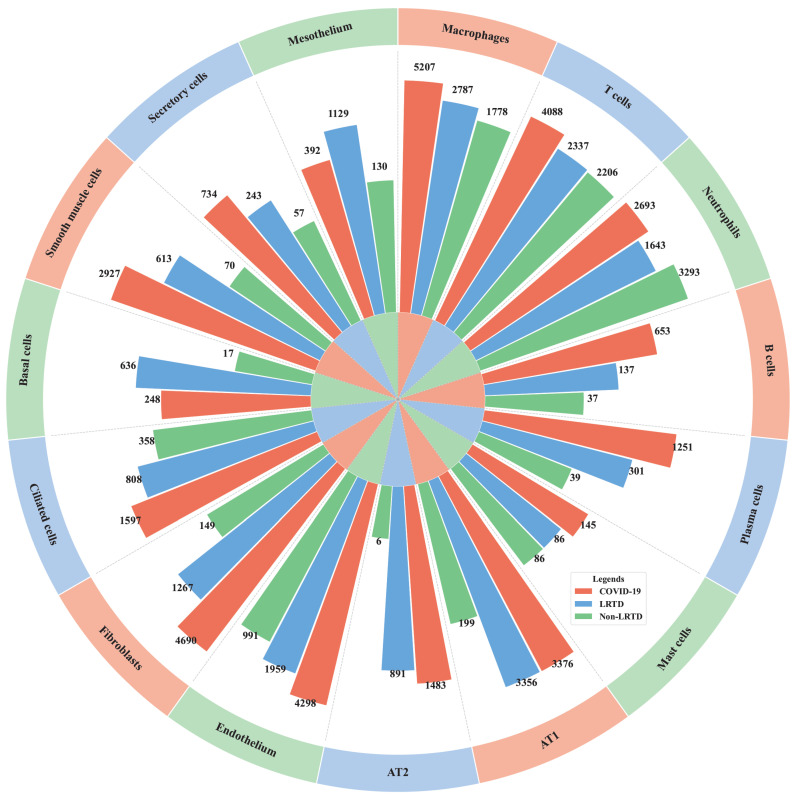
Cell type and status grouped donut chart. A logarithmic scale ring bar chart displays the classification and quantity of 15 cell types and their respective cellular states. Different sectors represent distinct cell types.

The data analyzed in this study were obtained from a spatially resolved single-cell transcriptomic study of lung tissues from Malawian patients [9] and are available through Zenodo (https://zenodo.org/records/13898423, accessed on 8 December 2024). The dataset comprised single-cell RNA sequencing profiles from lung autopsy specimens and included three disease groups: COVID-19 (patients with acute respiratory distress and confirmed SARS-CoV-2 infection at admission), LRTD (patients who presented with acute respiratory distress but remained consistently negative for SARS-CoV-2 throughout hospitalization), and non-LRTD (patients with no oxygen requirement or clinical evidence of LRTD and negative PCR results for SARS-CoV-2). Fifteen major cell types were represented, including alveolar type 1 epithelial cells (AT1), alveolar type 2 epithelial cells (AT2), basal cells, B cells, ciliated cells, endothelium, fibroblasts, macrophages, mast cells, mesothelium, neutrophils, plasma cells, secretory cells, smooth muscle cells, and T cells. Gene expression matrices were extracted separately for each cell type to identify disease signatures associated with specific cellular populations. Table 1 summarizes the 15 cell types and their distribution across 61,391 cells, and Figure 1 further illustrates dataset composition and the distribution of cell types across disease groups.

### 2.2. Outline of the Machine Learning-Based Framework

**Figure 2:**
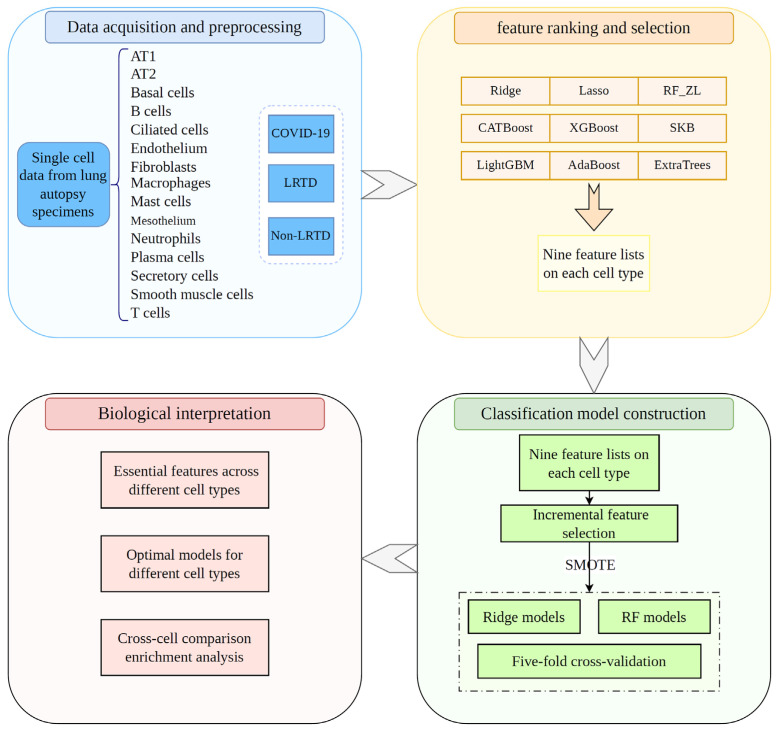
Flowchart of the machine learning-based framework. Single-cell transcriptomes from lung autopsy tissues were grouped into fifteen major cell types and labeled as COVID-19, LRTD, or Non-LRTD. Nine feature ranking algorithms were applied for evaluating features’ importance, generating nine feature lists per cell type. These lists were fed into an incremental feature selection method; lots of classification models using Ridge or random forest (RF) were constructed on balanced datasets processed by SMOTE. All models were evaluated by five-fold cross-validation. Finally, essential features, optimal models, and pathway-level biological interpretations were derived from cross-cell comparative analyses.

To identify cell-type-specific molecular signatures and systematically build robust disease-discrimination models, we applied an integrated ML-based framework. The overall procedure is illustrated in Figure 2. After obtaining the datasets described in Section 2.1, we used nine feature-ranking algorithms to evaluate gene importance from different statistical perspectives: Ridge Regression [12], Least Absolute Shrinkage and Selection Operator (Lasso) [13], Random Forest (RF_ZL) [14], Categorical Boosting (CATBoost) [15], EXtreme Gradient Boosting (XGBoost) [16], SelectKBest (SKB) [17], Light Gradient Boosting Machine (LightGBM) [18], Adaptive Boosting (AdaBoost) [19], and Extremely Randomized Trees (ExtraTrees) [20]. These algorithms were selected to comprehensively assess feature relevance by integrating linear, tree-based, and ensemble-learning approaches. Each ranking method generated a feature list for each cell type. We then applied the IFS method [10] to each list and built multiple classification models using Ridge Regression [12] or RF [14] on balanced datasets generated by SMOTE [11]. All models were evaluated by five-fold cross-validation [21]. It is necessary to point out that the SMOTE was only applied to the training set during each round of five-fold cross-validation so that the information of test samples was strictly isolated from the training procedure. For each feature list, the best-performing model was designated the optimal model, and the features used by that model were designated the optimal features. Biological interpretation was subsequently performed on these optimal features. Descriptions of the feature-ranking algorithms, classification algorithms, IFS procedure, and SMOTE are provided in File S1. The implementations of these ML algorithms were obtained from public resources and executed with default hyperparameters. Additional details are listed in Table S1.

### 2.3. Performance Metrics

To comprehensively evaluate the classification performance of the models generated during the IFS procedure, we used multiple metrics that account for the class imbalance inherent in the dataset.

#### 2.3.1. Weighted F1 Score

The weighted F1 score is a key metric for assessing model performance, especially in datasets with imbalanced class sizes [22,23,24]. Unlike the macro F1 score, which simply averages the F1 scores across classes, the weighted F1 score assigns weights according to class size so that larger classes contribute proportionally more to the overall score. This approach provides a more realistic assessment of model performance across classes of different sizes. The formulas used to calculate the weighted F1 score are as follows:

(1) $$Precision={\frac{{TP}_{i}}{{FP}_{i}+TP}}_{i}$$

(2) $${Precision}_{weighted}=\sum_{i=1}^{L}{Precision}_{i}\times \omega_{i}$$

(3) $${Recall}_{i}=\frac{{TP}_{i}}{{TP}_{i}+{FN}_{i}}$$

(4) $${Recall}_{weighted}=\sum_{i=1}^{L}{Recall}_{i}\times \omega_{i}$$

(5) $$Weighted\;F1\;score=\frac{2\times {Precision}_{weighted}\times {Recall}_{weighted}}{{Precision}_{weighted}+{Recall}_{weighted}}$$

Here, i denotes the i-th class (COVID-19, LRTD, or non-LRTD in this study), $\omega_{i}$ represents the proportion of samples in the i-th class relative to the total sample size, and L denotes the total number of classes. In addition, TP, FP, and FN indicate true positives, false positives, and false negatives, respectively. For each cell type, we used the weighted F1 score as the primary metric for selecting the optimal model from each feature list.

#### 2.3.2. Additional Performance Metrics

In addition to the weighted F1 score, we calculated two further metrics, accuracy (ACC) and the Matthews correlation coefficient (MCC) [25,26], to provide a more comprehensive evaluation of model performance. ACC measures the proportion of correctly classified samples. MCC is a balanced metric that simultaneously considers true positives, true negatives, false positives, and false negatives, and it is widely regarded as robust for imbalanced datasets.

### 2.4. Enrichment Analysis

To better interpret the identified genes in terms of function and pathway involvement, we performed a comprehensive Gene Ontology (GO) enrichment analysis using the gseapy package (version 1.1.10) and the Enrichr database [27,28]. The intersection genes for each cell type, identified through cross-algorithm union analysis, were tested against three GO databases: GO_Biological_Process, GO_Cellular_Component, and GO_Molecular_Function. Fisher’s exact test was used to assess enrichment significance, with false discovery rate correction for multiple testing; adjusted *p*-values < 0.05 were considered significant. Enriched terms were ranked using the combined score, calculated as the product of the log-transformed *p*-value and the z-score, to capture both biological relevance and statistical strength.

Pathway annotation was further conducted using KEGG enrichment with the gseapy enrichr function [29]. The discriminative gene sets from 15 cell types were queried against the KEGG_Human database. Fisher’s exact test with Benjamini–Hochberg correction was used for multiple comparisons, and an adjusted *p*-value threshold of 0.05 was applied. Enriched pathways were ranked by statistical significance and biological relevance to highlight key signaling cascades and metabolic processes that distinguish COVID-19 from other LRTDs.

## 3. Results

The workflow of this study is shown in Figure 2. We performed a comprehensive single-cell transcriptomic analysis of lung tissues from Malawian patients across three disease conditions: COVID-19, LRTDs, and non-LRTD controls. The analysis comprised four major stages: data acquisition and preprocessing, feature ranking and selection, classification model construction, and biological interpretation. Feature importance was evaluated using nine ranking algorithms, Ridge, Lasso, RF_ZL, CATBoost, XGBoost, SKB, LightGBM, AdaBoost, and ExtraTrees, yielding nine feature lists for each cell type. We then applied IFS with two classification algorithms (Ridge and RF) to each feature list to identify optimal models and features. Class imbalance during model training was addressed with SMOTE. GO enrichment analysis, KEGG pathway analysis, and detailed literature annotation were subsequently used to interpret the biological roles of the optimal genes in different disease states.

### 3.1. Feature Ranking Results

Nine feature-ranking algorithms were applied independently to the datasets for the 15 cell types, producing corresponding ranked feature lists. Higher-ranked features generally had greater potential biological relevance and were therefore of particular interest in subsequent analyses. The complete feature lists for all 15 cell types are provided in Table S2. However, key features could not be identified directly from these ranked lists alone, so further refinement was required. We therefore applied the IFS procedure to each list to determine which genes contributed positively and most strongly to model construction, as described in Section 2.2.

Because each list contained a very large number of features, we modified the original IFS procedure to improve computational efficiency. Specifically, only the top 2000 features were considered, as relatively few genes were expected to be highly informative for disease classification. In addition, we used a step size of five to construct feature subsets and models; that is, we first built a model using the top five features in a given list, then added the next five features to build the subsequent model, and so on. All models were evaluated by five-fold cross-validation, and macro F1 score, weighted F1 score, ACC, and MCC were recorded, as shown in Table S3. F1 curves were then plotted to provide a clearer view of model performance as the feature number increased, with the weighted F1 score on the y-axis and the number of features on the X-axis.

**Figure 3:**
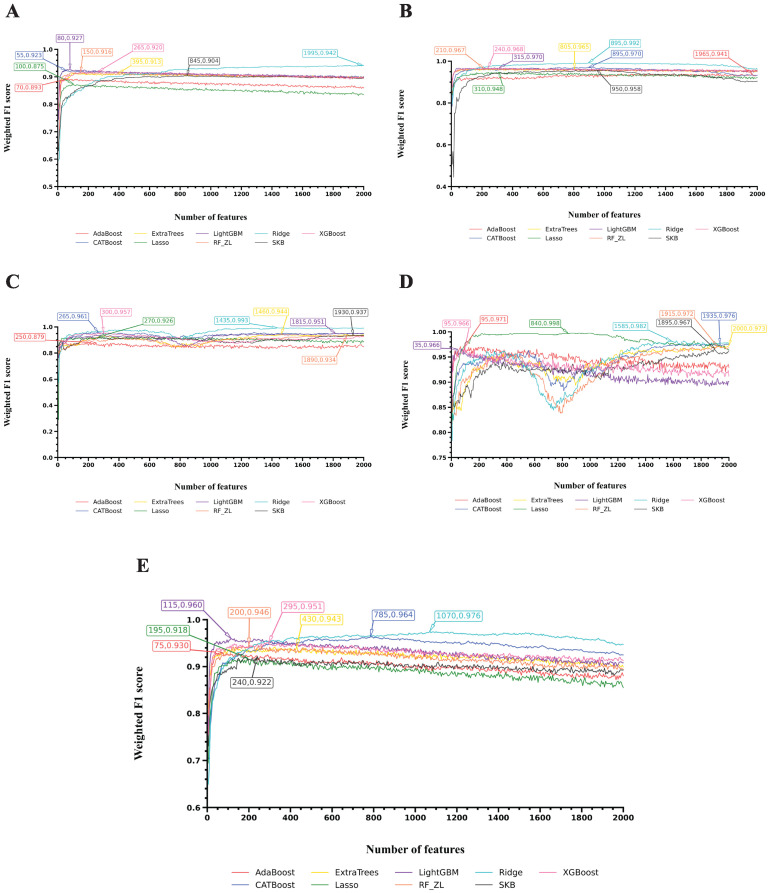
IFS curves for the classification algorithms used to build the optimal models on feature lists yielded by nine feature ranking algorithms. (**A**): IFS curve of AT1; (**B**): IFS curve of AT2; (**C**): IFS curve of Basal cells; (**D**): IFS curve of B cells; (**E**): IFS curve of Ciliated cells.

**Figure 4:**
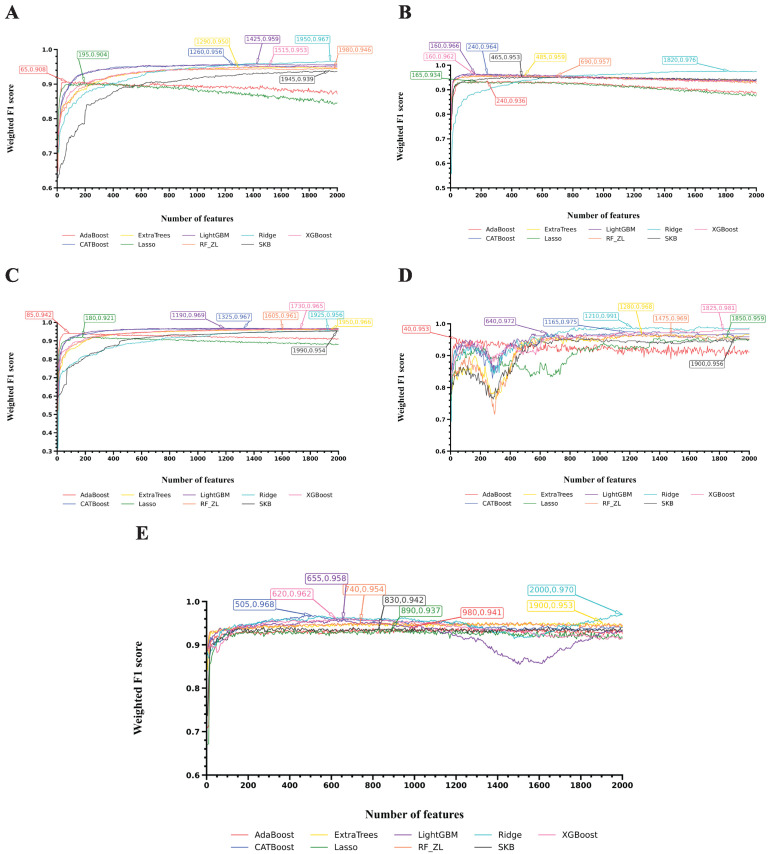
IFS curves for the classification algorithms used to build the optimal models on feature lists yielded by nine feature ranking algorithms. (**A**): IFS curve of Endothelium; (**B**): IFS curve of Fibroblasts; (**C**): IFS curve of Macrophages; (**D**): IFS curve of Mast cells; (**E**): IFS curve of Mesothelium.

**Figure 5:**
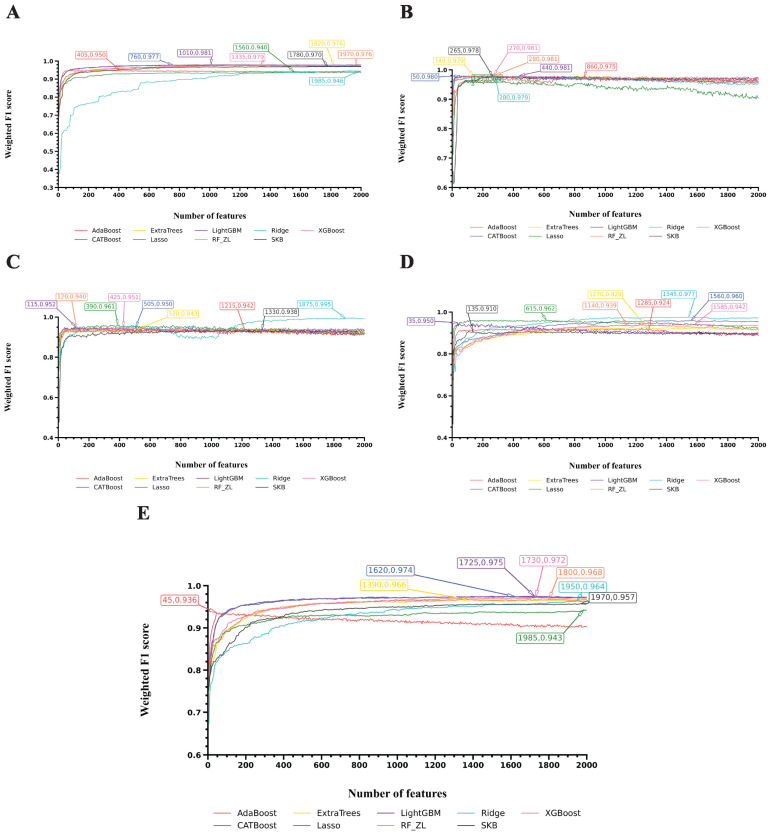
IFS curves for the classification algorithms used to build the optimal models on feature lists yielded by nine feature ranking algorithms. (**A**): IFS curve of Neutrophils; (**B**): IFS curve of Plasma cells; (**C**): IFS curve of Secretory cells; (**D**): IFS curve of Smooth muscle cells; (**E**): IFS curve of T cells.

**Figure 6:**
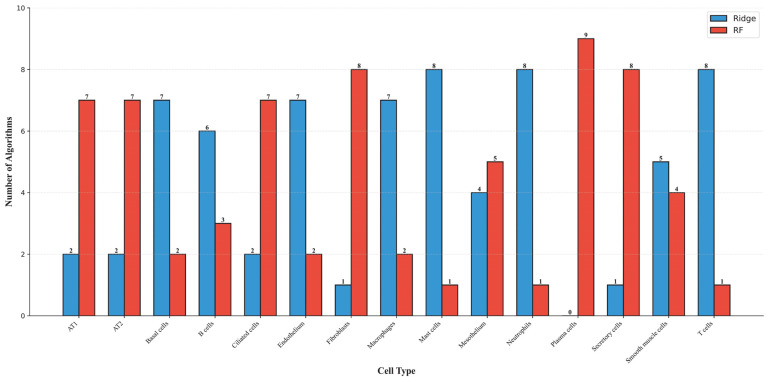
Bar chart displaying the number of Ridge and RF used to construct optimal models on nine feature lists across different cell types.

To visualize the performance of the optimal models for each cell type, we selected, for each case, the classifier that produced the highest weighted F1 score among Ridge and RF and plotted the corresponding F1 curves, as shown in Figure 3, Figure 4 and Figure 5. Different coordinate panels represent different cell types. Figure 6 summarizes the number of Ridge or RF that was selected to build the optimal models for each cell type, revealing a degree of cell-type-specific preference in algorithm selection. Detailed information on the chosen algorithm for each cell type is provided in Table 2.

Figure 3 presents the F1 curves for five cell types: AT1, AT2, Basal cells, B cells, and Ciliated cells. In four of these five cell types, the best performance was achieved using the feature list generated by Ridge, with the highest weighted F1 scores of 0.942 for AT1, 0.992 for AT2, 0.993 for Basal cells, and 0.976 for Ciliated cells. In contrast, the feature list generated by Lasso produced the best result for B cells, with a weighted F1 score of 0.998.

Figure 4 shows the F1 curves for another five cell types: Endothelium, Fibroblasts, Macrophages, Mast cells, and Mesothelium. Feature lists generated by Ridge again ranked highest for four cell types, yielding weighted F1 scores of 0.967 for Endothelium, 0.976 for Fibroblasts, 0.991 for Mast cells, and 0.970 for Mesothelium. For Macrophages, however, the feature list generated by LightGBM performed best, with a weighted F1 score of 0.969.

Figure 5 illustrates the F1 curves for the remaining five cell types: Neutrophils, Plasma cells, Secretory cells, Smooth muscle cells, and T cells. The feature list generated by Ridge performed best for two cell types, yielding weighted F1 scores of 0.995 for Secretory cells and 0.977 for Smooth muscle cells. For two other cell types, the best performance was obtained from feature lists generated by LightGBM, with weighted F1 scores of 0.981 for Neutrophils and 0.975 for T cells. Interestingly, for Plasma cells, feature lists generated by XGBoost, LightGBM, and RF_ZL all achieved the same optimal weighted F1 score of 0.981.

### 3.2. Cross-Algorithm Union Analysis

For each cell type, we identified optimal features from each feature list through the IFS procedure. Based on these features, Ridge or RF produced the highest weighted F1 score, indicating that the selected genes were informative for distinguishing COVID-19, LRTD, and non-LRTD samples. However, in many cases, very large numbers of optimal features were required to achieve the highest weighted F1 scores, which conflicted with our goal of identifying key genes with minimal redundancy. To improve the strategy and maximize the practical usefulness of our findings, we defined an elbow point, also referred to as an inflection point, on F1 curves that required many features to reach peak performance. This point was determined using a threshold-based method. The weighted F1 threshold was selected empirically by examining the IFS curves and was generally set slightly below the highest weighted F1 score; the first point exceeding this threshold usually required far fewer features than the full optimal set. This point was therefore considered the elbow point. Importantly, the model built with the features at the elbow point still maintained strong classification performance, indicating that these features were more essential than the remaining optimal features. These reduced sets were designated the final essential feature subsets for each cell type. It should also be noted that when the number of optimal features was not large, generally fewer than 100, no elbow point was defined. In such cases, the optimal features themselves were treated as essential features for consistency.

After essential features had been obtained from the nine feature lists for each cell type, we compared them across the different feature-ranking algorithms. The overlap patterns among these features are shown in Figure S1, which provides a comprehensive view of cross-algorithm agreement. Features identified repeatedly by multiple algorithms were reasonably considered more important and biologically relevant, because consistent selection across distinct methodological approaches suggests a fundamental role in disease classification. Table S4 lists the genes identified by one or more feature-ranking algorithms and constitutes the primary basis for subsequent functional enrichment analysis and biological interpretation.

### 3.3. Results of Enrichment Analysis

**Figure 7:**
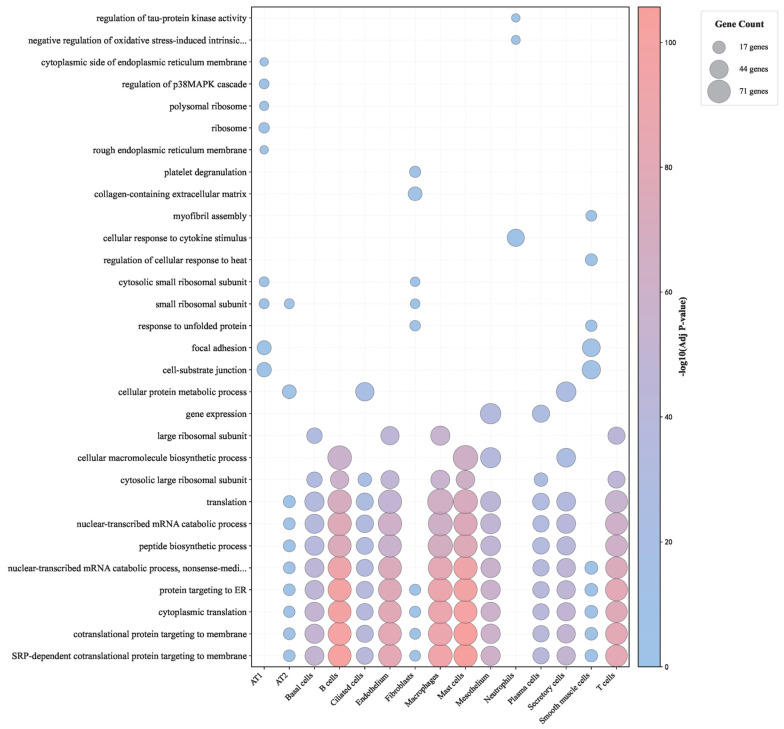
GO biological process enrichment across fifteen lung cell types in COVID-19. Bubble plot showing GO biological process enrichment (–log_10_ adjusted p value) across lung cell types. Bubble size denotes the number of enriched genes, and color represents enrichment significance. Translational and ribosome-related processes—such as peptide biosynthesis, cytoplasmic translation, and ribosomal subunit assembly—are consistently and strongly enriched across multiple cell types, highlighting broad activation of protein synthesis pathways in COVID-19. In contrast, stress-response, adhesion, and extracellular matrix-related processes exhibit more cell-type-specific enrichment patterns.

For each cell type, the essential genes identified by the different feature-ranking algorithms were combined and subjected to GO enrichment analysis, revealing both shared disease mechanisms and cell-type-specific molecular signatures. As shown in Figure 7, cross-cell-type analysis identified 30 pathways enriched across all cell types, highlighting fundamental molecular processes affected by COVID-19. These universally enriched pathways included cytoplasmic translation and peptide biosynthesis, neutrophil degranulation and activation, platelet aggregation, nuclear-transcribed mRNA catabolism, cellular response to unfolded protein, and regulation of cell death and inflammatory signaling. In contrast, cell-type-specific enrichment patterns pointed to functional specialization: immune cells showed strong enrichment in activation and cytokine-production pathways, alveolar epithelial cells showed stress responses and surfactant metabolic alterations, and endothelial cells displayed signatures related to vascular remodeling and permeability regulation. Detailed results are provided in Table S5.

**Figure 8:**
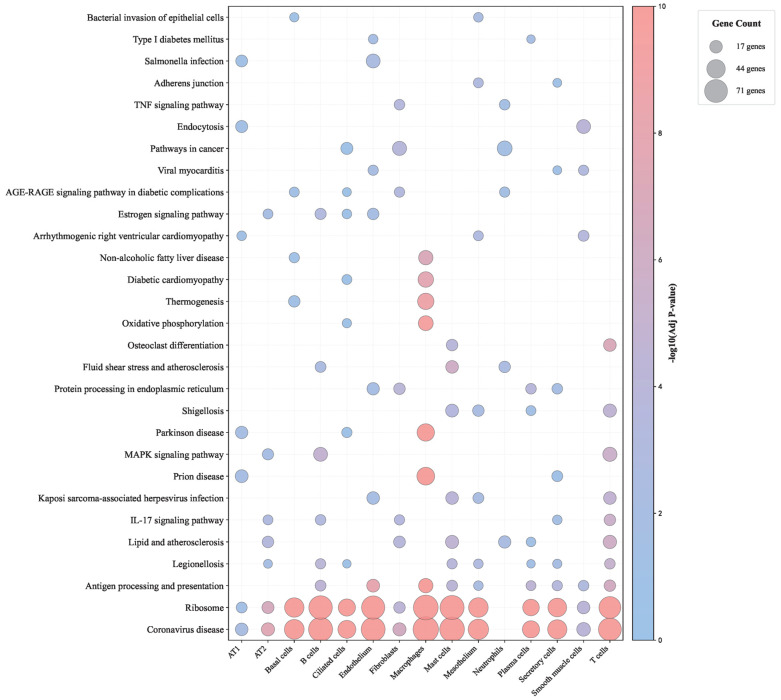
KEGG pathway enrichment across fifteen lung cell types in COVID-19. Bubble plot showing KEGG pathway enrichment (–log_10_ adjusted p value) across lung cell types. Bubble size indicates the number of enriched genes, and color reflects the enrichment significance. Coronavirus disease, ribosome, IL-17 signaling, and antigen presentation pathways show strong and broad enrichment in COVID-19, whereas metabolic and signaling pathways display more cell-type-specific patterns.

KEGG pathway enrichment analysis was also performed on the essential genes across the 15 cell types, identifying 375 significantly enriched pathways among the 3503 pathways examined (Figure S2). The top 10 pathways for each cell type were selected for comparison and visualized in a bubble chart (Figure 8), and detailed results are provided in Table S6. The analysis revealed multiple pathways significantly associated with COVID-19 across cell types (FDR < 0.05). The coronavirus disease pathway was enriched in 14 cell types, with the strongest enrichment observed in AT2 cells (*p* = 3.73 × 10^−8^) and fibroblasts (*p* = 4.12 × 10^−7^), indicating that SARS-CoV-2-specific response programs were broadly activated across diverse lung compartments. Immune-related pathways also showed cell-type-specific patterns. For example, the antigen processing and presentation pathway was enriched in nine cell types, including professional antigen-presenting cells as well as structural cells such as endothelium and mesothelium, whereas the IL-17 signaling pathway was enriched in five cell types, reflecting proinflammatory responses. Enrichment of the Legionellosis pathway in eight cell types suggested shared host–pathogen interaction mechanisms between bacterial and viral respiratory infections, while enrichment of the lipid and atherosclerosis pathway in six cell types indicated metabolic dysregulation and potential vascular complications characteristic of severe COVID-19.

## 4. Discussion

Following the comprehensive analysis of single-cell transcriptomic data from patients with COVID-19 and other LRTDs, we identified essential genes (features) using multiple feature-ranking algorithms.

### 4.1. Functional Enrichment Reveals Convergent and Divergent Pathological Programs

Our functional enrichment analysis provides mechanistic context for the ML-identified gene signatures and clarifies how COVID-19 differs from other LRTDs at the pathway level. Three major findings emerge from this analysis.

First, the widespread enrichment of protein synthesis and cellular stress-response pathways across multiple cell types points to core disruptions in cellular proteostasis during SARS-CoV-2 infection. This observation is consistent with the coronavirus replication cycle, in which host translation programs are extensively reprogrammed to support viral protein synthesis [30,31,32]. Notably, these translation-related disturbances may occur not only in infected cells but also in bystander cells exposed to paracrine inflammatory signals, which may explain why translation-associated genes emerged as ML-identified discriminative features across diverse cell types, including cells not directly infected with SARS-CoV-2 [33].

Second, the analysis revealed broadly shared immune pathway enrichment across both immune and structural cells, including neutrophil degranulation, macrophage activation, and antigen presentation pathways. These findings suggest that COVID-19 immunopathology is systemic rather than confined to canonical immune cell compartments. The presence of these pathways in both immune and structural cells indicates that structural cells may play more active roles than traditionally assumed. This broader activation pattern may reflect intercellular crosstalk and may help explain why ML-identified features in these cells, such as NFKBIA in neutrophils and HLA-DQA2 in macrophages, contribute to cell-type-specific immunopathology. Moreover, MHC II expression in nonclassical antigen-presenting cells during severe SARS-CoV-2 infection has been reported previously and has been linked to persistent inflammation and cytokine storm phenotypes [34].

Third, the IFN-γ-associated macrophage signature highlighted in the original Malawian cohort differs from the type I/III interferon-dominant patterns reported in some US, European, and Asian cohorts. This contrast provides an important context for interpreting the ML-identified transcriptional features in the Malawian dataset. It suggests that genetic background, endemic infections such as HIV and malaria, and prior environmental exposures may have shaped host responses to SARS-CoV-2 and produced alternative immunological trajectories that could require different therapeutic approaches. The potential contribution of parasitic infections to the alternative activation of resident macrophages, as well as interference in antiviral responses associated with HIV coinfection, may help explain the IFN-γ response pattern observed in this African population [35,36,37].

Importantly, the enrichment profile identified coronavirus disease pathways across 15 cell types, alongside cell-type-specific patterns, such as surfactant metabolism in AT2 cells and IL-17 responses in neutrophils, supporting the biological relevance of our model rather than suggesting technical or housekeeping artifacts. The enrichment of pathways related to Legionellosis and bacterial infections across multiple cell types further suggests that bacterial and viral respiratory infections share elements of host–pathogen interaction and common cellular stress responses, despite differences in disease etiology. Overall, these pathway-level findings not only validate the identified gene sets but also point to potential therapeutic avenues, including interventions tailored to population-specific responses, restoration of alveolar repair pathways, and correction of aberrant immune activation in structural cells.

### 4.2. Cell-Type-Specific Molecular Signatures and Pathological Mechanisms

We focused on alveolar epithelial cells, innate immune cells, and adaptive immune cells because these populations represent the principal pathological interfaces in respiratory viral infection. The alveolar epithelial cell types AT1 and AT2 are the primary sites of viral entry and tissue injury, making their gene expression profiles particularly informative for understanding geographically specific patterns of lung damage in COVID-19. Innate immune cells, mainly macrophages and neutrophils, orchestrate the early inflammatory response and showed some of the clearest population-specific patterns in the Malawian dataset, making them especially valuable for understanding geographically distinct disease manifestations. Adaptive immune cells, represented here by T and B cells, influence either disease resolution or the development of severe immunopathology. Their disturbances in SARS-CoV-2 infection differ markedly from those reported in bacterial pneumonia and other LRTDs.

Importantly, the genes discussed below were drawn from the intersection of essential genes identified by multiple feature-ranking algorithms, as summarized in Table S4. These genes were selected as representative examples because of their strong discriminatory performance and are used here to illustrate cell-type-specific functional alterations and immune response characteristics within the infected microenvironment.

#### 4.2.1. Alveolar Epithelial Cell Injury, Dysfunctional Regeneration, and Metabolic Alterations

As the major site of gas exchange and a direct point of viral entry, the alveolar epithelium undergoes profound alterations in COVID-19. CAV1, a marker of mature AT1 cells, emerged as a key feature for AT1-cell classification across four algorithms. Previous studies have shown that both the number of AT1 cells and the expression of AT1 marker genes are substantially reduced in patients with COVID-19. Consistent with this pattern, CAV1 mRNA expression remained markedly reduced in AT1 cells from patients with COVID-19 relative to healthy controls, potentially reflecting severe epithelial injury and inefficient AT1-cell maturation and repair [38]. In addition, MALAT1, a long noncoding RNA, and several mitochondria-related genes (MT-CO3, MT-ND3, and MT-ATP6) showed increased expression, indicating heightened stress responses, altered cellular metabolism, and apoptotic activity. These genes were also identified as important feature-ranking genes by multiple algorithms [39,40]. Their elevated expression further supports an association with lung injury and inflammation in severe COVID-19. These changes may promote epithelial cell apoptosis and the release of chemoattractants such as IL-8 in neutrophils, thereby reinforcing a cycle of alveolar inflammation and injury [39]. By comparison, MALAT1 and mitochondria-related genes may be less affected in other LRTDs, potentially contributing to a more regulated inflammatory response.

In COVID-19, AT2 cells show markedly increased expression of SFTPC and EVL, suggesting arrest in a transitional state. They also exhibit stress-related and proinflammatory transcriptional programs, including significant induction of epithelium-derived IL-6. This excessive regenerative response may paradoxically impair the efficient maturation of AT2 cells into fully functional epithelial cells [41]. In other LRTDs, AT2 cells may retain a greater capacity to transdifferentiate into AT1 cells. In addition, dysregulated expression of AZGP1, which contributes to immune and metabolic regulation, and NPC2, which is involved in lipid—especially cholesterol—transport during COVID-19, points to abnormal surfactant metabolism in alveolar spaces, a proposed hallmark of AT2 cell dysfunction in COVID-19 [42,43].

#### 4.2.2. Innate Immune Cells: Unique IFN-γ-Driven Macrophage Response and Neutrophil Dysregulation

Our source data show that alveolar macrophages (AMs) in the COVID-19 group highly express tissue-resident macrophage markers, including the complement-related genes C1QC and C1QB, and display strong activation of inflammatory pathways centered on type II interferon (IFN-γ). These features suggest a role in both immune-mediated epithelial injury and antiviral clearance. According to the original Malawian cohort study, this macrophage-associated IFN-γ response differs from the type I/III interferon-related patterns reported in some non-African cohorts. The same study also found that diffuse lung injury in the LRTD group was dominated by neutrophil-mediated fibrinopurulent inflammation, whereas COVID-19 exhibited more heterogeneous inflammatory patterns [9].

Single-cell transcriptomic analyses have shown that, relative to other LRTDs, macrophages in the lungs of patients with COVID-19 display marked differences in the expression of several key genes. In COVID-19, alveolar macrophages tend to express high levels of CD163 and MRC1, which encodes the CD206 receptor, suggesting a prominent M2-like, alternatively activated polarization state [44]. In contrast, macrophages in other pulmonary infections, including non-COVID pneumonias, more often exhibit a classically activated proinflammatory phenotype, frequently with high expression of acute inflammatory genes such as S100A8 and S100A9 [45]. In the IFN-γ-rich environment observed in COVID-19, macrophages also show stronger antigen-presentation signals, including upregulation of MHC class II molecules such as HLA-DQA2 and HLA-DRB5, consistent with findings from our source dataset [9]. Single-cell studies of severe COVID-19 have likewise identified a macrophage population with high CD163 and MRC1 expression, further supporting an M2-polarized phenotype [44].

Neutrophils are first responders in pneumonia, and their behavior in COVID-19 versus other LRTDs highlights fundamental differences in immune response. In typical bacterial pneumonia or acute respiratory distress syndrome (ARDS), neutrophils dominate the early inflammatory phase, rapidly migrating into alveolar spaces to phagocytose pathogens and release granule enzymes. This response is usually acute and localized. In COVID-19, however, neutrophils not only infiltrate the lungs, contributing to diffuse alveolar damage and microvascular obstruction, but also remain persistently activated and display abnormal immature phenotypes in the circulation. Patients with severe COVID-19 often exhibit neutrophilia with circulating immature forms such as band cells and low-density neutrophils, phenomena that are uncommon in conventional pneumonia [46]. In our analysis, NFKBIA and IL1R2 were identified by multiple algorithms as important discriminative markers in neutrophils. Previous studies have shown that neutrophils in COVID-19 upregulate inflammatory regulators such as NFKBIA and IL1R2, suggesting a feedback attempt to restrain excessive inflammatory signaling. In particular, elevated IL1R2 expression may reflect neutrophil responses to excessive IL-1 and may represent a protective mechanism against pyroptosis or tissue injury. LITAF was identified by eight ranking algorithms as a key feature, likely reflecting its role in pathogen killing by neutrophils in LRTDs [47]. During bacterial infection, neutrophils strongly express LITAF (LPS-induced TNFα factor), which promotes TNFα production and the release of enzymes such as myeloperoxidase and elastase that can cause local tissue damage. Although these mechanisms help contain infection, they may also contribute to alveolar damage, manifesting as consolidation in bacterial pneumonia. Our analysis also identified NAMPT as an inflammatory mediator produced by both neutrophils and lung cells that can enhance neutrophil survival and cytokine release. Activation of NAMPT-associated pathways has also been reported in severe COVID-19 [48].

#### 4.2.3. Adaptive Immune Cells: Hyperactivation, Exhaustion, and Enhanced Plasmablast Differentiation

Although innate immune cells dominate the early phase of acute lung infection, adaptive immune cells, including T and B lymphocytes, also play critical roles in determining disease progression. One of the most striking differences between COVID-19 and other forms of pneumonia lies in the functional state of T cells. In typical community-acquired pneumonia, T-cell responses are generally effective, supporting pathogen clearance and the generation of immune memory [49]. Even when transient lymphopenia occurs, T-cell numbers usually recover quickly. In contrast, severe COVID-19 is often associated with marked T-cell dysfunction and sustained lymphopenia, and CD4^+^ and CD8^+^ T cells in peripheral blood are significantly reduced, often showing evidence of exhaustion or activation-induced apoptosis [50,51]. Single-cell transcriptomic studies have shown that T cells in patients with COVID-19 display characteristics that differ from those seen in other LRTDs. Viral pneumonias such as COVID-19 are marked by the expansion of cytotoxic effector T cells with high expression of cytolytic molecules such as GNLY and GZMB [52]. In influenza and other LRTDs, CD8^+^ effector T_EMRA cells with high GNLY expression are also commonly observed in peripheral blood. However, in COVID-19, T cells additionally exhibit a broader and more intense type I interferon-stimulated gene (ISG) program, including notable upregulation of MHC class I molecules such as HLA-B and B2M, which helps distinguish COVID-19 T-cell responses from those in other pulmonary infections [52]. Moreover, T cells in COVID-19 show a distinctive pattern of hyperactivation and proliferation. In severe cases, FOS and JUN, members of the AP-1 transcription factor family, are significantly upregulated in pulmonary CD4^+^ T cells, indicating a higher activation state than that typically observed in LRTDs [53]. Meanwhile, the long noncoding RNA MALAT1 is markedly downregulated in proliferating T cells from patients with COVID-19, a pattern that has been validated as a hallmark of T-cell proliferation [54]. Another notable finding is the rapid downregulation of KLF2, a transcription factor involved in maintaining T-cell quiescence and migration, reflecting a broad shift from a resting to an effector state in COVID-19 [55]. Collectively, genes such as GNLY, FOS/JUN, and MALAT1 serve as informative single-cell markers distinguishing T-cell responses in COVID-19 from those in other respiratory infections.

In COVID-19 lung infection, B cells also exhibit a highly activated transcriptional state skewed toward plasmablast differentiation, a pattern that differs from that seen in other pneumonias. In patients with COVID-19, ELL2 is significantly upregulated in B cells, indicating marked plasmablast expansion in severe disease accompanied by a sharp increase in immunoglobulin transcripts. This feature is not commonly observed in other LRTDs [56]. The regulatory long noncoding RNA MALAT1 is also markedly downregulated in B cells and other immune cells during severe COVID-19, a change that is uncommon in typical bacterial or viral pneumonias [57]. This downregulation may be related to the heightened inflammatory environment in COVID-19, which is less pronounced in other LRTDs. In addition, B cells in COVID-19 show immediate-early gene and stress-response patterns similar to those observed in T cells, further distinguishing them from B cells in other pneumonias. For example, the AP-1 family transcription factors FOSB and JUN are strongly induced in COVID-19 B cells as part of the acute proinflammatory response. Likewise, upregulation of heat shock proteins such as HSPA1A reflects the cellular stress experienced within the inflamed pulmonary environment [58]. These genes are often transiently elevated during severe SARS-CoV-2 infection and mark a highly inflammatory, antibody-secreting B-cell state that represents a distinctive immune feature of COVID-19.

In summary, our study integrates single-cell transcriptomic data with ML approaches to systematically compare gene expression profiles and immune responses across multiple lung cell types in Malawian patients with COVID-19 and other LRTDs. The findings highlight COVID-19-associated transcriptional signatures of cellular injury and immune microenvironmental alteration, particularly in alveolar epithelial cells, innate immune cells, and T/B lymphocytes. These observations provide a basis for future validation of regionally relevant biomarkers and disease hypotheses.

### 4.3. Limitations of This Study

Several limitations should also be acknowledged in current study. First, this study is a reanalysis of the single-cell dataset reported by Nyirenda et al., and the identified signatures should therefore be interpreted as associative rather than causal. Second, the biological interpretations derived from ML-based feature selection and GO/pathway enrichment analyses primarily generate hypotheses for further investigation and do not, by themselves, establish functional mechanisms. Although these findings may help characterize disease-associated transcriptional features, they should not be overinterpreted as evidence of universal biological mechanisms or direct therapeutic targets. Further validation in independent cohorts and functional studies will be necessary to confirm the biological and clinical relevance of these observations. Third, the dataset includes only 30 patients, and the sample size is limited, with substantial imbalance across disease groups and cell types. The identified biomarkers should therefore be interpreted with caution. They may provide useful clues about single-cell expression patterns, but additional experimental validation of candidate biomarker genes is still needed. Finally, the ML-based framework was not very rigorous in some aspects. For example, default hyperparameters were adopted to execute ML algorithms in the framework, inducing the suboptimal results. Furthermore, we empirically selected the threshold for identifying elbow point. This operation lacked objectivity. Some false positive genes may be included or the actual essential genes were discarded.

## 5. Conclusions

This study provides a computational analysis of single-cell RNA sequencing profiles from lung autopsy specimens using an ML-based framework. The framework generated strong classification models that achieved robust disease discrimination (weighted F1 scores > 0.94) across 15 lung cell types while highlighting an IFN-γ-dominant macrophage response in Malawian patients. This finding suggests that interferon-related responses may vary across cohorts and should be interpreted in a population- and study-context-dependent manner. The identified cell-type-specific signatures, CAV1 downregulation in AT1 cells, SFTPC dysregulation in AT2 cells, and NFKBIA upregulation in neutrophils, along with the universal disruption of protein synthesis machinery, provide mechanistic insight into why COVID-19 produces pathological outcomes that differ from those of other respiratory infections despite shared clinical features. Our incremental feature selection approach further shows that parsimonious gene signatures can maintain high predictive accuracy, offering practical value for diagnostic biomarker development in resource-limited settings. Overall, this work underscores that molecular disease mechanisms cannot be assumed to be universal across populations. Future validation in larger African cohorts and functional studies of divergent interferon responses will be important for developing equitable precision medicine strategies that account for pathogenic diversity across human populations.

## Figures and Tables

**Table 1:** Summary of lung cell types and their distribution across different disease groups.

Cell Types	Number of Cells on Different Disease Groups		
	COVID-19	LRTD	Non-LRTD
Macrophages	5207	2787	1778
T cells	4088	2337	2206
Neutrophils	2693	1643	3293
B cells	653	137	37
Plasma cells	1251	301	39
Mast cells	145	86	86
AT1	3376	3356	199
AT2	1483	891	6
Endothelium	4298	1959	991
Fibroblasts	4690	1267	149
Ciliated cells	1597	808	358
Basal cells	248	636	17
Smooth muscle cells	2927	613	70
Secretory cells	734	243	57
Mesothelium	392	1129	130

**Table 2:** Classification algorithm used to construct the optimal model for each cell type across different feature ranking algorithms.

Cell Type	Feature Ranking Algorithm								
	AdaBoost	CATBoost	ExtraTrees	Lasso	LightGBM	RF_ZL	Ridge	SKB	XGBoost
AT1	RF	RF	RF	RF	RF	RF	Ridge	Ridge	RF
AT2	RF	Ridge	RF	RF	RF	RF	Ridge	RF	RF
Basal cells	RF	Ridge	Ridge	RF	Ridge	Ridge	Ridge	Ridge	Ridge
B cells	RF	Ridge	Ridge	Ridge	RF	Ridge	Ridge	Ridge	RF
Ciliated cells	RF	Ridge	RF	RF	RF	RF	Ridge	RF	RF
Endothelium	RF	Ridge	Ridge	RF	Ridge	Ridge	Ridge	Ridge	Ridge
Fibroblasts	RF	RF	RF	RF	RF	RF	Ridge	RF	RF
Macrophages	RF	Ridge	Ridge	RF	Ridge	Ridge	Ridge	Ridge	Ridge
Mast cells	RF	Ridge	Ridge	Ridge	Ridge	Ridge	Ridge	Ridge	Ridge
Mesothelium	RF	Ridge	RF	RF	Ridge	RF	Ridge	RF	Ridge
Neutrophils	RF	Ridge	Ridge	Ridge	Ridge	Ridge	Ridge	Ridge	Ridge
Plasma cells	RF	RF	RF	RF	RF	RF	RF	RF	RF
Secretory cells	RF	RF	RF	RF	RF	RF	Ridge	RF	RF
Smooth muscle cells	RF	Ridge	Ridge	RF	RF	Ridge	Ridge	RF	Ridge
T cells	RF	Ridge	Ridge	Ridge	Ridge	Ridge	Ridge	Ridge	Ridge

## Data Availability

The data analyzed in this study is available at Zenodo (https://zenodo.org/records/13898423, accessed on 8 December 2024). The analyzed results are contained within the article or Supplementary Materials.

## Supplementary Materials

The following supporting information can be downloaded at: https://www.mdpi.com/article/10.3390/life16050771/s1, File S1: Descriptions on used machine learning algorithms; Table S1: Details of machine learning algorithms used in this study. Table S2: Feature lists yielded by nine feature ranking algorithms; Table S3: Performance evaluation of two classification algorithms on nine feature lists based on IFS method; Table S4: Intersection of nine essential gene sets identified by AdaBoost, CATboost, ExtraTrees, Lasso, LightGBM, RF_ZL, Ridge, SKB and XGBoost; Table S5: Essential genes and GO enrichment summary. This table lists, for each lung cell type, the number of essential genes identified and the number of significantly enriched GO terms (FDR < 0.05). These results summarize the functional characteristics associated with the essential gene sets identified in the study; Table S6: Essential genes and KEGG enrichment summary. This table lists, for each lung cell type, the number of essential genes identified and the number of significantly enriched KEGG pathways (FDR < 0.05). These results summarize the major biological pathways associated with the essential gene sets identified in this study; Figure S1: The UpSet chart displaying the essential feature subsets identified by different feature ranking algorithms and their intersections. The top bar chart shows the size of the intersections of various feature subsets, while the horizontal bar chart on the left displays the sizes of the feature subsets themselves. The main grid consists of dots and connecting lines, where black dots indicate which algorithms are part of each intersection set. Gray dots represent algorithms not included in a particular intersection. Figure S2: Pathway enrichment analysis across different cell types and tissue samples. Top enriched pathways in fifteen cell types. Bar plots showing significantly enriched biological pathways (*p* < 0.05, dashed line) for AT1, AT2, basal cells, B cells, ciliated cells, endothelium, fibroblasts, macrophages, mast cells, mesothelium, neutrophils, plasma cells, secretory cells, smooth muscle cells and T cells. X-axis: −log_10_(Adjusted *p*-value); longer bars indicate higher significance. Colors: blue (most significant) to orange/red (less significant). Numbers in parentheses indicate total significant pathways per cell type. Common enriched pathways include protein synthesis, translation, cytoplasmic processes, and RNA/DNA metabolism.

## Abbreviations

The following abbreviations are used in this manuscript:

COVID-19	Coronavirus Disease 2019
LRTD	Lower respiratory tract disease
ARDS	Acute respiratory distress syndrome
SARS-CoV-2	severe acute respiratory syndrome coronavirus 2
ML	Machine learning
IFS	Incremental feature selection
SMOTE	Synthetic Minority Oversampling Technique
AT1	Alveolar type 1 epithelial cell
AT2	Alveolar type 2 epithelial cell
Lasso	Least Absolute Shrinkage and Selection Operator
CATBoost	Categorical Boosting
XGBoost	EXtreme Gradient Boosting
SKB	SelectKBest
LightGBM	Light Gradient Boosting Machine
AdaBoost	Adaptive Boosting
ExtraTrees	Extremely Randomized Trees
MCC	Matthews Correlation Coefficient

## References

[1] Liu P., Xu M., Cao L., Su L., Lu L., Dong N., Jia R., Zhu X., Xu J. (2021). Impact of COVID-19 pandemic on the prevalence of respiratory viruses in children with lower respiratory tract infections in china. Virol. J..

[2] Lu S., Huang X., Liu R., Lan Y., Lei Y., Zeng F., Tang X., He H. (2022). Comparison of COVID-19 induced respiratory failure and typical ards: Similarities and differences. Front. Med..

[3] Giamarellos-Bourboulis E.J., Netea M.G., Rovina N., Akinosoglou K., Antoniadou A., Antonakos N., Damoraki G., Gkavogianni T., Adami M.-E., Katsaounou P. (2020). Complex immune dysregulation in COVID-19 patients with severe respiratory failure. Cell Host Microbe.

[4] Wauters E., Van Mol P., Garg A.D., Jansen S., Van Herck Y., Vanderbeke L., Bassez A., Boeckx B., Malengier-Devlies B., Timmerman A. (2021). Discriminating mild from critical COVID-19 by innate and adaptive immune single-cell profiling of bronchoalveolar lavages. Cell Res..

[5] Wang S., Yao X., Ma S., Ping Y., Fan Y., Sun S., He Z., Shi Y., Sun L., Xiao S. (2021). A single-cell transcriptomic landscape of the lungs of patients with COVID-19. Nat. Cell Biol..

[6] Xiao K., Cao Y., Han Z., Zhang Y., Luu L.D.W., Chen L., Yan P., Chen W., Wang J., Liang Y. (2025). A pan-immune panorama of bacterial pneumonia revealed by a large-scale single-cell transcriptome atlas. Signal Transduct. Target. Ther..

[7] Sorensen R., Barber R., Pigott D., Carter A., Spencer C., Ostroff S., Reiner R., Abbafati C., Adolph C., Allorant A. (2022). Variation in the COVID-19 infection-fatality ratio by age, time, and geography during the pre-vaccine era: A systematic analysis. Lancet.

[8] Okonji E.F., Okonji O.C., Mukumbang F.C., Van Wyk B. (2021). Understanding varying COVID-19 mortality rates reported in africa compared to europe, americas and asia. Trop. Med. Int. Health.

[9] Nyirenda J., Hardy O.M., Silva Filho J.D., Herder V., Attipa C., Ndovi C., Siwombo M., Namalima T.R., Suwedi L., Ilia G. (2024). Spatially resolved single-cell atlas unveils a distinct cellular signature of fatal lung COVID-19 in a malawian population. Nat. Med..

[10] Liu H., Setiono R. (1998). Incremental feature selection. Appl. Intell..

[11] Chawla N., Bowyer K., Hall L.O., Kegelmeyer W.P. (2002). Smote: Synthetic minority over-sampling technique. arXiv.

[12] Hoerl A.E., Kennard R.W. (1970). Ridge regression: Biased estimation for nonorthogonal problems. Technometrics.

[13] Tibshirani R. (1996). Regression shrinkage and selection via the lasso. J. R. Stat. Soc. Ser. B (Methodol.).

[14] Breiman L. (2001). Random forests. Mach. Learn..

[15] Dorogush A.V., Ershov V., Gulin A. (2018). Catboost: Gradient boosting with categorical features support. arXiv.

[16] Chen T., Guestrin C. (2016). Xgboost: A scalable tree boosting system. Proceedings of the 22nd ACM SIGKDD International Conference on Knowledge Discovery and Data Mining, San Francisco, CA, USA, 13–17 August 2016.

[17] Ayyanar M., Jeganathan S., Parthasarathy S., Jayaraman V., Lakshminarayanan A.R. (2022). Predicting the cardiac diseases using selectkbest method equipped light gradient boosting machine. Proceedings of the 2022 6th International Conference on Trends in Electronics and Informatics (ICOEI), Tirunelveli, India, 2022.

[18] Ke G., Meng Q., Finley T., Wang T., Chen W., Ma W., Ye Q., Liu T.-Y. (2017). Lightgbm: A highly efficient gradient boosting decision tree. Adv. Neural Inf. Process. Syst..

[19] Freund Y., Schapire R.E. (1997). A decision-theoretic generalization of on-line learning and an application to boosting. J. Comput. Syst. Sci..

[20] Geurts P., Ernst D., Wehenkel L. (2006). Extremely randomized trees. Mach. Learn..

[21] Kohavi R. (1995). A study of cross-validation and bootstrap for accuracy estimation and model selection. Proceedings of the International Joint Conference on Artificial Intelligence, Montreal Quebec, Canada, 20–25 August 1995.

[22] Powers D.M. (2020). Evaluation: From precision, recall and f-measure to roc, informedness, markedness and correlation. arXiv.

[23] Ren J., Gao Q., Zhou X., Feng K., Guo W., Huang T., Cai Y.-D. (2026). Identification of gene signatures differentiating cancer from normal tissues across histological classifications of gastric adenocarcinoma via machine learning methods. Biochem. Genet..

[24] Chen L., Xun X., Zhou B. (2026). Root-associated protein prediction using a protein large language model and hypergraph convolutional networks. Sci. Rep..

[25] Matthews B. (1975). Comparison of the predicted and observed secondary structure of t4 phage lysozyme. Biochim. Biophys. Acta (BBA)-Protein Struct..

[26] Gorodkin J. (2004). Comparing two k-category assignments by a k-category correlation coefficient. Comput. Biol. Chem..

[27] Fang Z., Liu X., Peltz G. (2023). Gseapy: A comprehensive package for performing gene set enrichment analysis in python. Bioinformatics.

[28] Ashburner M., Ball C.A., Blake J.A., Botstein D., Butler H., Cherry J.M., Davis A.P., Dolinski K., Dwight S.S., Eppig J.T. (2000). Gene ontology: Tool for the unification of biology. The gene ontology consortium. Nat. Genet..

[29] Kanehisa M., Goto S. (2000). Kegg: Kyoto encyclopedia of genes and genomes. Nucleic Acids Res..

[30] Finkel Y., Gluck A., Nachshon A., Winkler R., Fisher T., Rozman B., Mizrahi O., Lubelsky Y., Zuckerman B., Slobodin B. (2021). SARS-CoV-2 uses a multipronged strategy to impede host protein synthesis. Nature.

[31] Eriani G., Martin F. (2022). Viral and cellular translation during SARS-CoV-2 infection. FEBS Open Bio.

[32] Zhang D., Zhu L., Wang Y., Li P., Gao Y. (2022). Translational control of COVID-19 and its therapeutic implication. Front. Immunol..

[33] Patra T., Ray R. (2022). Bystander effect of SARS-CoV-2 spike protein on human monocytic thp-1 cell activation and initiation of prothrombogenic stimulus representing severe COVID-19. J. Inflamm..

[34] Vanderbeke L., Van Mol P., Van Herck Y., De Smet F., Humblet-Baron S., Martinod K., Antoranz A., Arijs I., Boeckx B., Bosisio F.M. (2021). Monocyte-driven atypical cytokine storm and aberrant neutrophil activation as key mediators of COVID-19 disease severity. Nat. Commun..

[35] Jang J.C., Nair M.G. (2013). Alternatively activated macrophages revisited: New insights into the regulation of immunity, inflammation and metabolic function following parasite infection. Curr. Immunol. Rev..

[36] Hume D.A. (2015). The many alternative faces of macrophage activation. Front. Immunol..

[37] Sandstrom T.S., Ranganath N., Angel J.B. (2017). Impairment of the type i interferon response by hiv-1: Potential targets for hiv eradication. Cytokine Growth Factor Rev..

[38] Lamers M.M., Haagmans B.L. (2022). SARS-CoV-2 pathogenesis. Nat. Rev. Microbiol..

[39] Abbasi-Kolli M., Nahand J.S., Kiani S.J., Khanaliha K., Khatami A.R., Taghizadieh M., Torkamani A.R., Babakhaniyan K., Bokharaei-Salim F. (2022). The expression patterns of malat-1, neat-1, thril, and mir-155-5p in the acute to the post-acute phase of COVID-19 disease. Braz. J. Infect. Dis..

[40] Duan C., Ma R., Zeng X., Chen B., Hou D., Liu R., Li X., Liu L., Li T., Huang H. (2022). SARS-CoV-2 achieves immune escape by destroying mitochondrial quality: Comprehensive analysis of the cellular landscapes of lung and blood specimens from patients with COVID-19. Front. Immunol..

[41] Melms J.C., Biermann J., Huang H., Wang Y., Nair A., Tagore S., Katsyv I., Rendeiro A.F., Amin A.D., Schapiro D. (2021). A molecular single-cell lung atlas of lethal COVID-19. Nature.

[42] de Andrade J.R., de Farias J.B., de Lima Vitorino M.L., Travassos F.T., da Silva R.A., de Lima Filho J.L., Valença M.M. (2025). Cerebrospinal fluid proteomic profiling reveals proteins associated with neuroinflammatory response in COVID-19 patients. ACS Omega.

[43] La Rosa P., Tiberi J., Palermo E., Tiano S., Cortese M., Hiscott J., Fiorenza M.T. (2023). Inactivation of the niemann pick c1 cholesterol transporter 1 (npc1) restricts SARS-CoV-2 infection. bioRxiv.

[44] Zhu A., Zhou L., Chen Z., Liu D., Feng H., Cai B., Chen X., Zhao J., Zhao J., Chen J. (2024). Single-cell analysis reveals t cell dysfunction driven by macrophages and differential expression of transposable elements in severe COVID-19 patients. Heliyon.

[45] Liao M., Liu Y., Yuan J., Wen Y., Xu G., Zhao J., Cheng L., Li J., Wang X., Wang F. (2020). Single-cell landscape of bronchoalveolar immune cells in patients with COVID-19. Nat. Med..

[46] Dwivedi A., Mhaonaigh A.U., Carroll M., Khosravi B., Batten I., Ballantine R.S., Phelan S.H., O’Doherty L., George A.M., Sui J. (2024). Emergence of dysfunctional neutrophils with a defect in arginase-1 release in severe COVID-19. JCI Insight.

[47] Chhabra R., Ball C., Chantrey J., Ganapathy K. (2018). Differential innate immune responses induced by classical and variant infectious bronchitis viruses in specific pathogen free chicks. Dev. Comp. Immunol..

[48] Roberts K. (2012). Regulation of Neutrophil Function by Nampt. Ph.D. Thesis.

[49] Eddens T., Kolls J.K. (2012). Host defenses against bacterial lower respiratory tract infection. Curr. Opin. Immunol..

[50] Chen Z., John Wherry E. (2020). T cell responses in patients with COVID-19. Nat. Rev. Immunol..

[51] Jarjour N.N., Masopust D., Jameson S.C. (2021). T cell memory: Understanding COVID-19. Immunity.

[52] Schuurman A.R., Reijnders T.D., Saris A., Ramirez Moral I., Schinkel M., de Brabander J., van Linge C., Vermeulen L., Scicluna B.P., Wiersinga W.J. (2021). Integrated single-cell analysis unveils diverging immune features of COVID-19, influenza, and other community-acquired pneumonia. eLife.

[53] Kalfaoglu B., Almeida-Santos J., Tye C.A., Satou Y., Ono M. (2020). T-cell hyperactivation and paralysis in severe COVID-19 infection revealed by single-cell analysis. Front. Immunol..

[54] Dey S., Ashwin H., Milross L., Hunter B., Majo J., Filby A.J., Fisher A.J., Kaye P.M., Lagos D. (2023). Downregulation of malat1 is a hallmark of tissue and peripheral proliferative t cells in COVID-19. Clin. Exp. Immunol..

[55] Jha P., Das H. (2017). Klf2 in regulation of nf-κb-mediated immune cell function and inflammation. Int. J. Mol. Sci..

[56] Kim C.W., Oh J.E., Lee H.K. (2021). Single cell transcriptomic re-analysis of immune cells in bronchoalveolar lavage fluids reveals the correlation of b cell characteristics and disease severity of patients with SARS-CoV-2 infection. Immune Netw..

[57] Huang K., Wang C., Vagts C., Raguveer V., Finn P.W., Perkins D.L. (2022). Long non-coding rnas (lncrnas) neat1 and malat1 are differentially expressed in severe COVID-19 patients: An integrated single-cell analysis. PLoS ONE.

[58] García-Vega M., Llamas-Covarrubias M.A., Loza M., Reséndiz-Sandoval M., Hinojosa-Trujillo D., Melgoza-González E., Valenzuela O., Mata-Haro V., Hernández-Oñate M., Soto-Gaxiola A. (2024). Single-cell transcriptomic analysis of b cells reveals new insights into atypical memory b cells in COVID-19. J. Med. Virol..

