# Supplementary material for: Machine Learning Identification of Cell-Type-Specific Molecular Signatures Distinguishing COVID-19 from Other Lower Respiratory Tract Diseases
Source: Life (Basel). 2026 May 4;16(5):771. doi: 10.3390/life16050771 (PMC13208634; doi:10.3390/life16050771)
Supplement: Supplementary file 1 [file life-16-00771-s001.zip › life-4219234-supplementary/File S1.pdf]

File S1. Descriptions on used machine learning algorithms

1. Classification Feature Ranking Algorithms

1.1. Ridge Regression

Ridge Regression is a regularization method for linear regression models. As opposed to ordinary least squares (OLS), which only minimizes the sum of squared residuals, Ridge Regression minimizes the sum of squared residuals and an L2 penalty term, which equals the square of the magnitude of the coefficients multiplied by a tuning parameter (λ). This penalty term compels the model to balance between fitting the training data effectively and keeping the coefficients small, which leads to more stable and reliable predictions on new and unseen data. Ridge Regression can give us feature importance. The idea is that the size of the coefficients in a Ridge model, after normalizing the features to the same scale, reflects their contribution to the target variable. The larger the absolute value of a feature's coefficient, the more effect it has on the prediction, and the more important it is.

1.2 Random Forest

Random Forest makes the prediction by training an abundance of decision trees such that every tree is grown on some random set of training data and on some random set of features—a method referred to as "bagging." At the time of test, each tree in the "forest" votes, and the output predicted is the majority vote (classification) or average (regression) over trees.

One method of computing the relevance of features in Random Forests is to compute the average decrease in node impurity across the full forest of trees, typically with the Gini impurity or the mean squared error. A feature with bigger average decrease in impurity across the full forest is deemed to be more relevant.

2. Feature Ranking Algorithm

2.1 Lasso

Lasso (Least Absolute Shrinkage and Selection Operator) is the linear regression regularization technique that also offers the option of automated feature selection. The Lasso employs an L1 penalty term, which equals the absolute values of the model's coefficient sum. The automated feature selection is done by setting the model's irrelevant or redundant feature's coefficient to precisely zero. The absolute coefficient value of the non-zero coefficient measures the relative feature effect on the target prediction - the higher the absolute coefficient of the feature, the more significant it is (assuming all features at the identical scale are standardized).

2.2 CATboost

CATboost is an open-source library of gradient boosting developed by Yandex and optimized particularly for categorical features. One of the most widely used metrics for determining the importance of features in CATboost is the Loss Function Change. CatBoost randomizes the values in the given feature in the data and records the loss function increase that follows (typically an increase) to gauge the importance of the feature. The larger the loss increase is, the more critical the feature is in making accurate

predictions. The underlying principle of this is that if the feature is valuable, randomizing the values in the feature would greatly inhibit the model's performance.

2.3 XGBoost

XGBoost is an highly optimized, scalable, and end-to-endlibrarythat applies the gradient boosting framework. The average gain (or gain) is the typical measure used in XGBoost to quantify the importance of features. The average gain indicates the average improvement in the model's accuracy, i.e., the overall loss function reduction (e.g., mean squared error or log loss), that can be attributed to each feature in all the splits of all trees. The higher the total gain of the feature, the more important it is.

2.4 SKB

SKB is a simple yet effective univariate feature selection method that works by choosing the K most significant features in a dataset based on the strength with which each feature independently correlates with the target variable. It does this by applying a particular statistical test - e.g., chi-square for categorical targets, ANOVA F-value in the situation of regression, or mutual information for different types of data - to each feature individually and giving them scores based on how well they are correlated to the target. The algorithm then gives ranks to all features depending on these scores and keeps only the first K of them and throws away the rest.

2.5 LightGBM

LightGBM is a highly efficient and distributed gradient boosting framework developed by Microsoft, designed for fast training, low memory usage, and high performance on large-scale datasets. The most straightforward metric for assessing feature importance in LightGBM is the weight (also called frequency). This simply counts the number of times a feature is used to split data across all trees in the model. A feature used more often for splitting is considered more important.

2.6 AdaBoost

AdaBoost is the seminal and influential ensemble learning technique. Its essence lies in aggregating numerous weak learners—the most common choice of which are highly simplistic models such as shallow decision trees, or stumps—to become a strong classifier. Feature importance calculation most frequently utilized by the algorithm, is through the sum of the total error reduction of the model associated with each time the feature makes any split. Those features most often chosen for splits and providing the greatest reductions of the error, have higher importance scores.

2.7 ExtraTrees

ExtraTrees is an ensemble learning technique similar to the Random Forest algorithm but comes with an extra level of randomness, thereby encouraging the growth of even more varied and decorrelated trees. Ranking the importance of features of Extra Trees predominantly makes use of the mean decrease of impurity. The importance of any specific feature is computed by adding the total (or average) reduction of impurity by the feature across all the trees of the ensemble. If the feature produces a notable reduction of impurity, the feature is considered of high importance.

3. Incremental Feature Selection

IFS is a wrapper-based feature selection technique designed to uncover a compact set of informatively rich features from a training data set. The algorithm typically starts by ranking all features by some predefined criterion, e.g., the importance score of the features. Starting from the null set, features are added to the model stepwise, ordered by their importance ranking. After each addition, the quality of the model is evaluated through the use of a validation set or cross-validation method. An optimal set of features typically results at the intersection where the quality peaks or settles.

4. Synthetic Minority Oversampling Technique

SMOTE is one of the widely used algorithms that has especially been employed to address class imbalance - the examples of one class are considerably larger or smaller in quantity compared to others, which can lead to rampant bias in the prediction model. Unlike simple oversampling, which merely replicates minority class examples and can lead to overfitting, SMOTE generates artificial examples. The algorithm operates by repeatedly selecting randomly one instance from the minority class, identifying its k-nearest neighbors (also members of the minority class) and then creating a synthetic instance along the line segment between the source instance and one of its k-nearest neighbors. Above procedures are conducted several rounds until the size of minority class is equal to the size of majority class.
