# Supplementary material for: Machine Learning Identification of Cell-Type-Specific Molecular Signatures Distinguishing COVID-19 from Other Lower Respiratory Tract Diseases
Source: Life (Basel). 2026 May 4;16(5):771. doi: 10.3390/life16050771 (PMC13208634; doi:10.3390/life16050771)
Supplement: Supplementary file 1 [file life-16-00771-s001.zip › life-4219234-supplementary/Table S1.pdf]

Table S1. Details of machine learning algorithms used in this study.

Algorithm	Functions in this study	URL or platform
Least Absolute Shrinkage and Selection Operator	Feature ranking	Scikit-learn
Categorical Boosting	Feature ranking	https://catboost.ai/en/docs/concepts/installation
EXtreme Gradient Boosting	Feature ranking	https://xgboost.readthedocs.io/en/stable/
Adaptive Boosting	Feature ranking	Scikit-learn
Extremely Randomized Trees	Feature ranking	Scikit-learn
Light Gradient Boosting Machine	Feature ranking	https://lightgbm.readthedocs.io/en/latest/
SelectKBest	Feature ranking	Scikit-learn
Random Forest	Feature ranking, Classification	Scikit-learn
Ridge Regression	Feature ranking, Classification	Scikit-learn
Synthetic Minority Over-sampling Technique	Dataset balanced	https://github.com/scikitlearn-contrib/imbalanced-learn
