# Supplementary figures and images for: Machine Learning Identification of Cell-Type-Specific Molecular Signatures Distinguishing COVID-19 from Other Lower Respiratory Tract Diseases
Source: Life (Basel). 2026 May 4;16(5):771. doi: 10.3390/life16050771 (PMC13208634; doi:10.3390/life16050771)

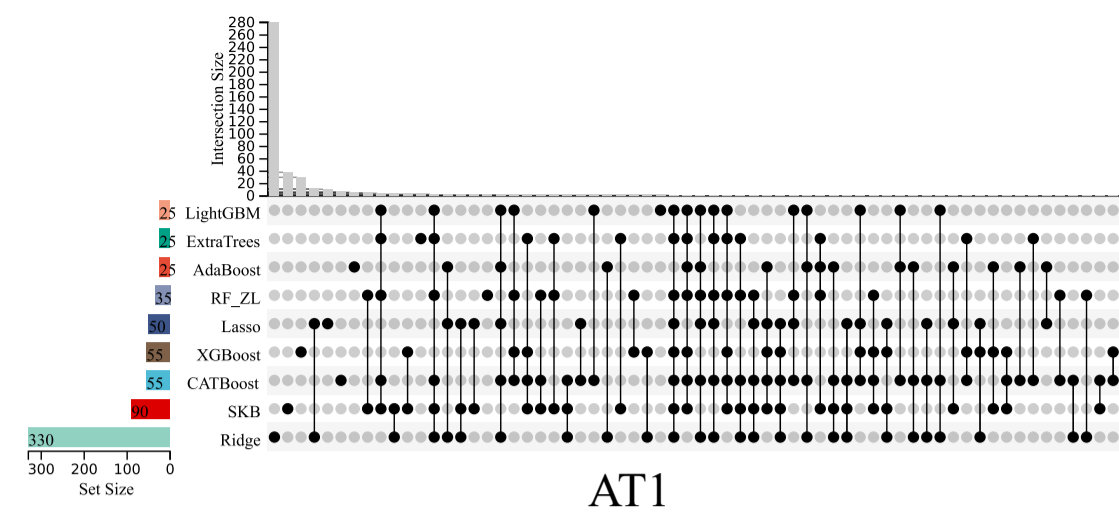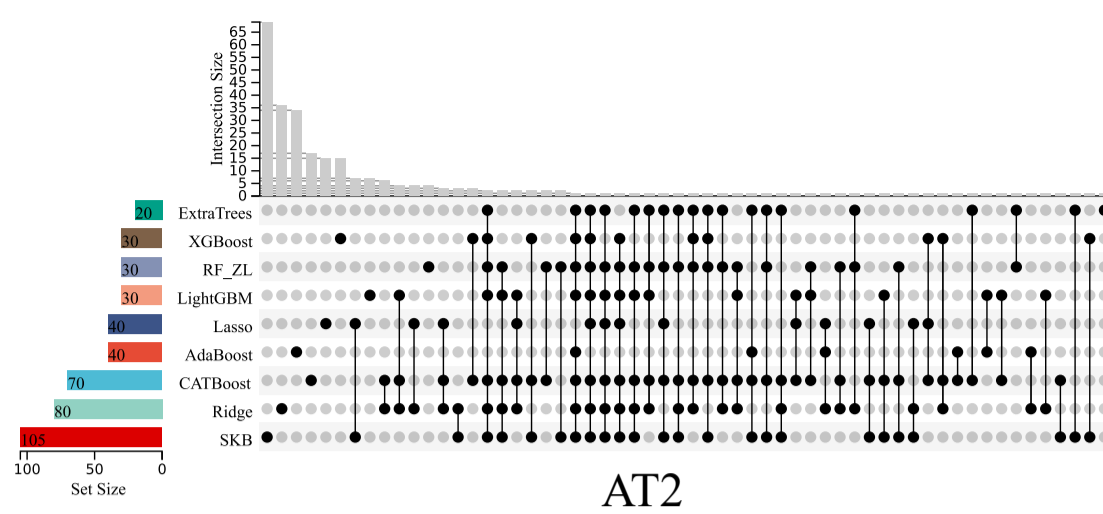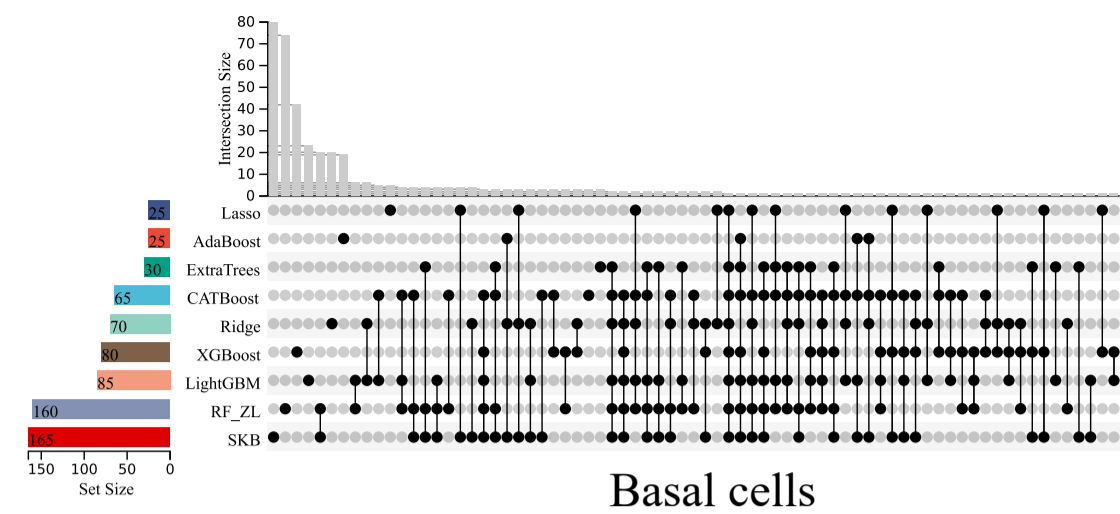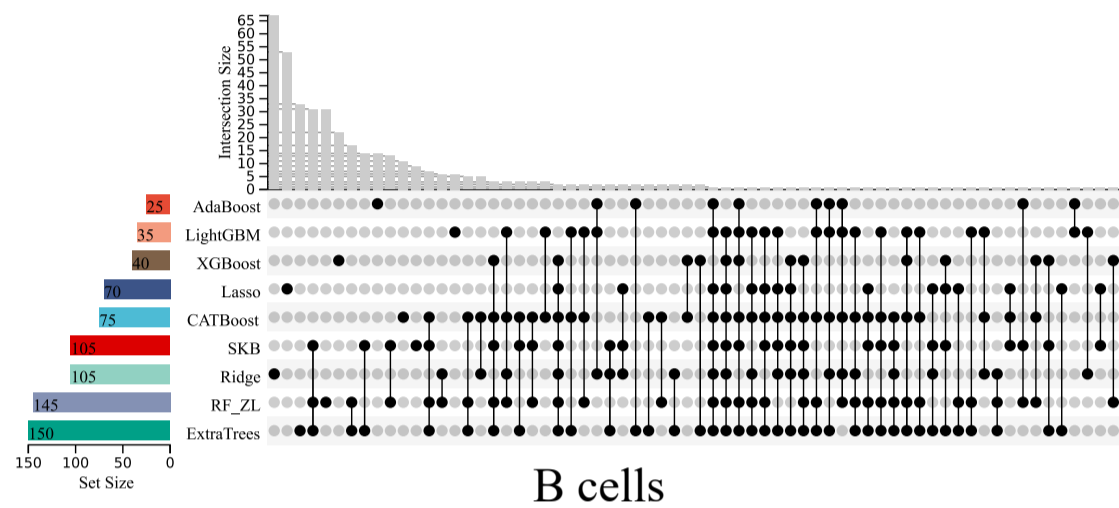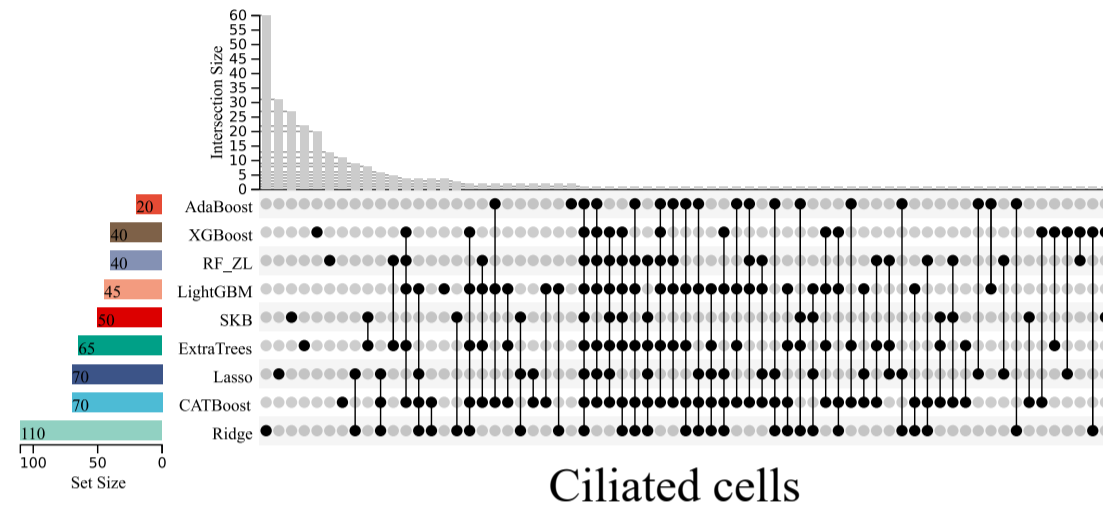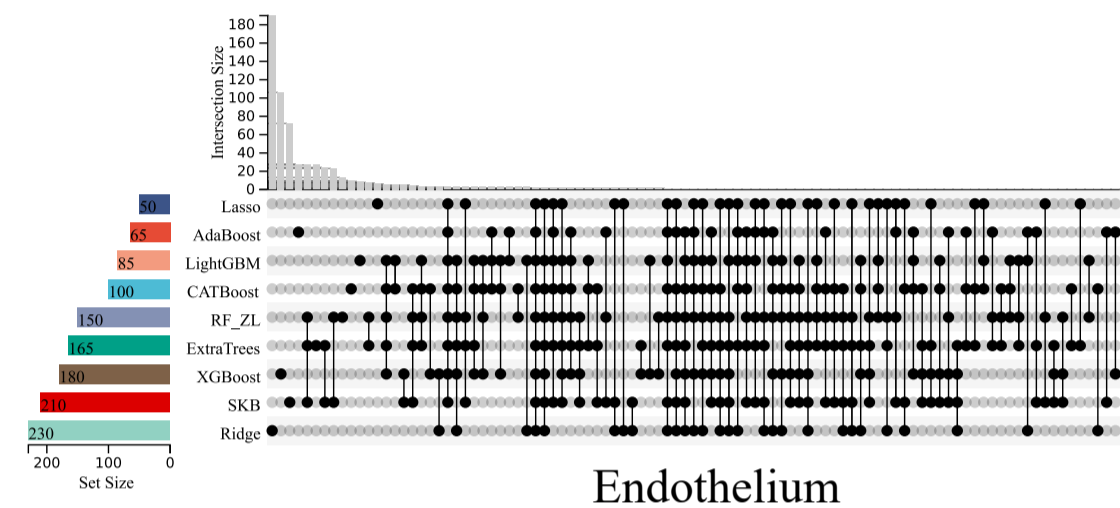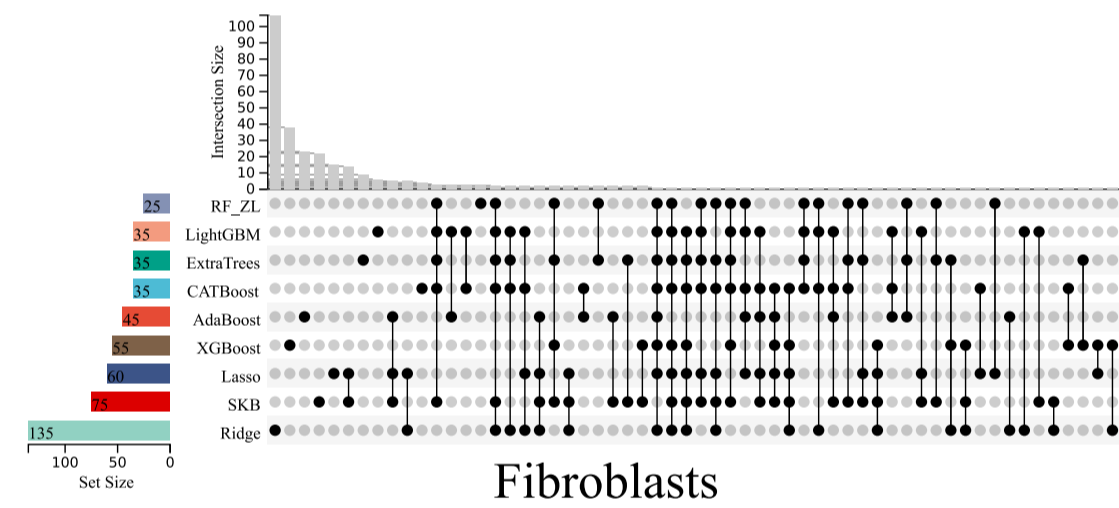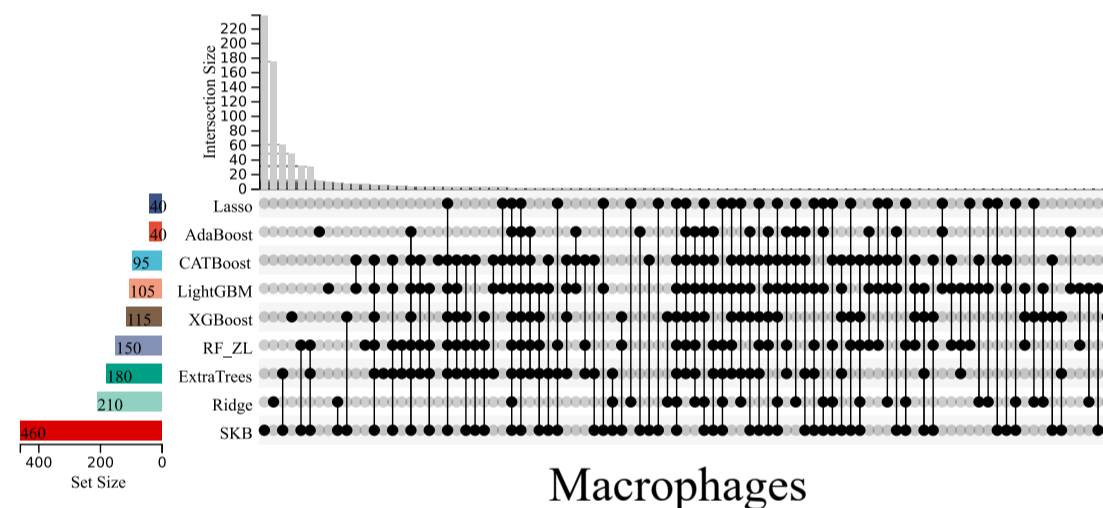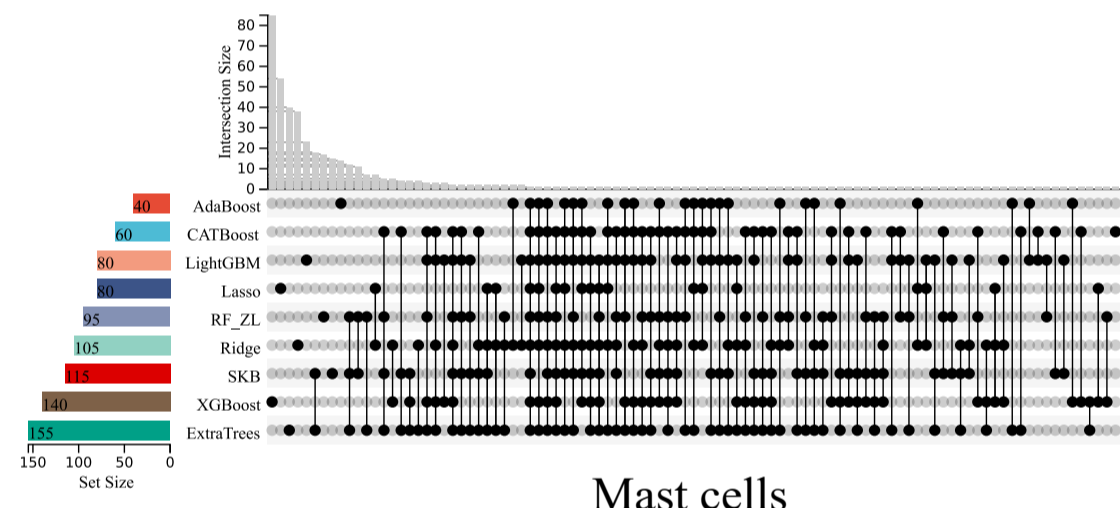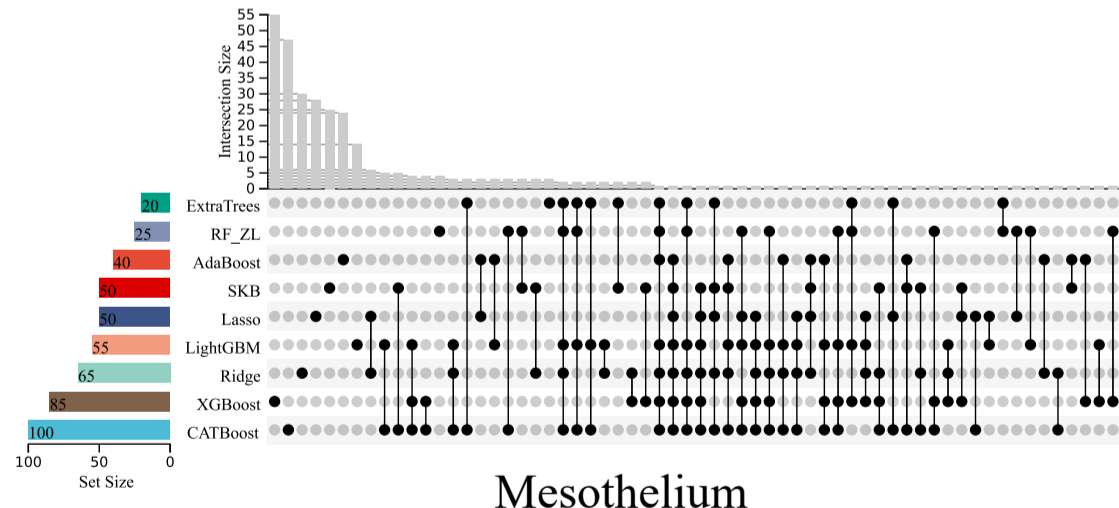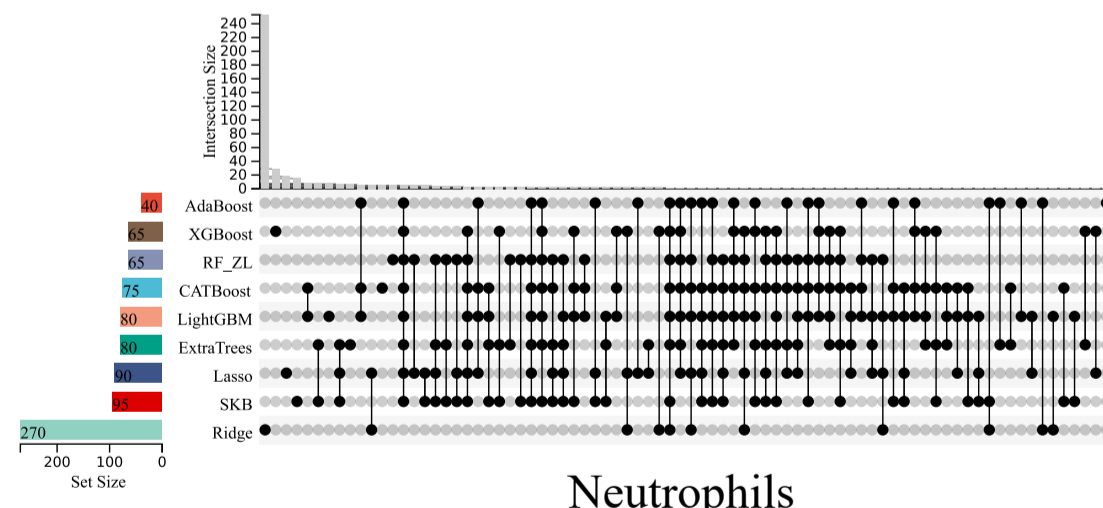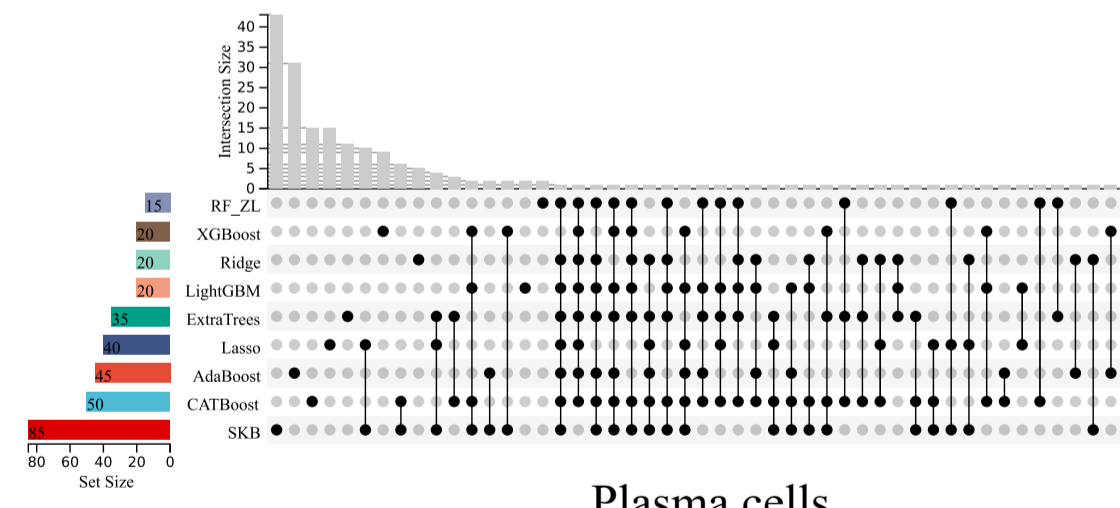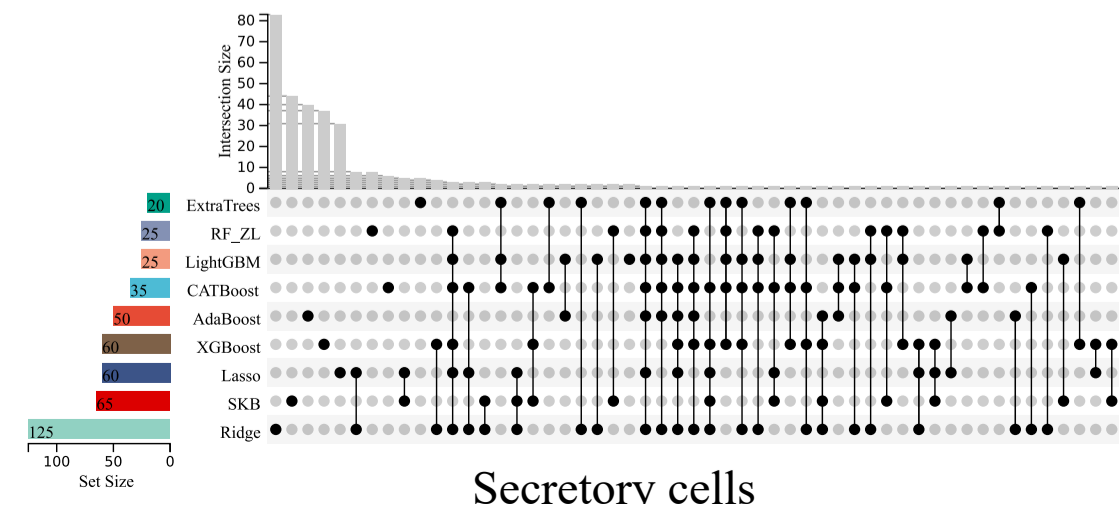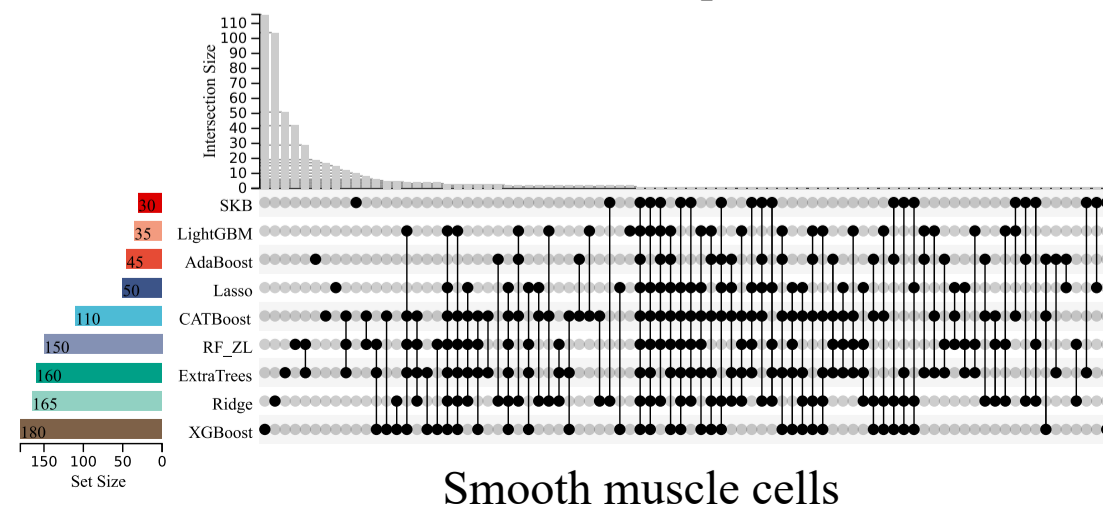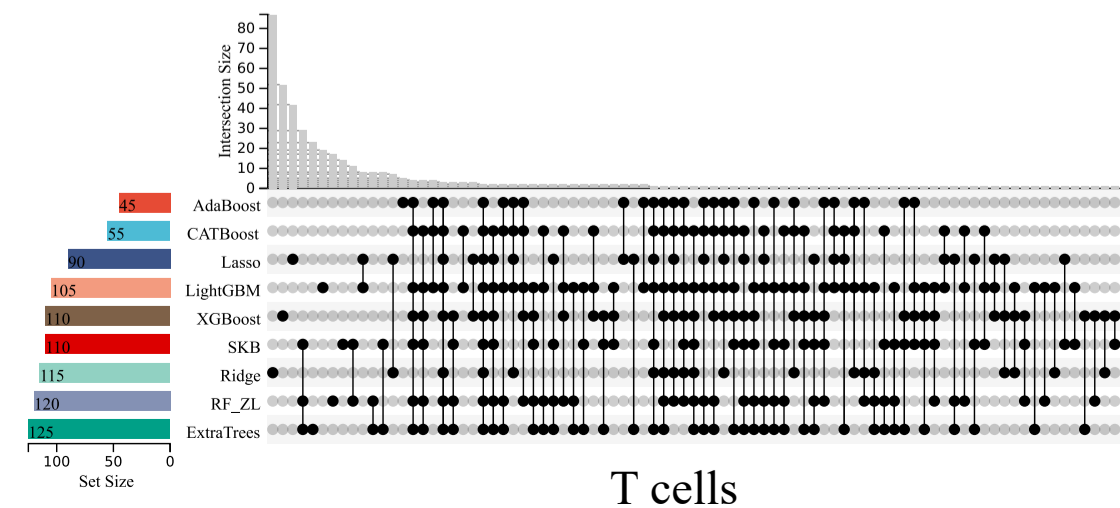

Supplement: Supplementary file 1 [file life-16-00771-s001.zip › life-4219234-supplementary/Figure S1.pdf]

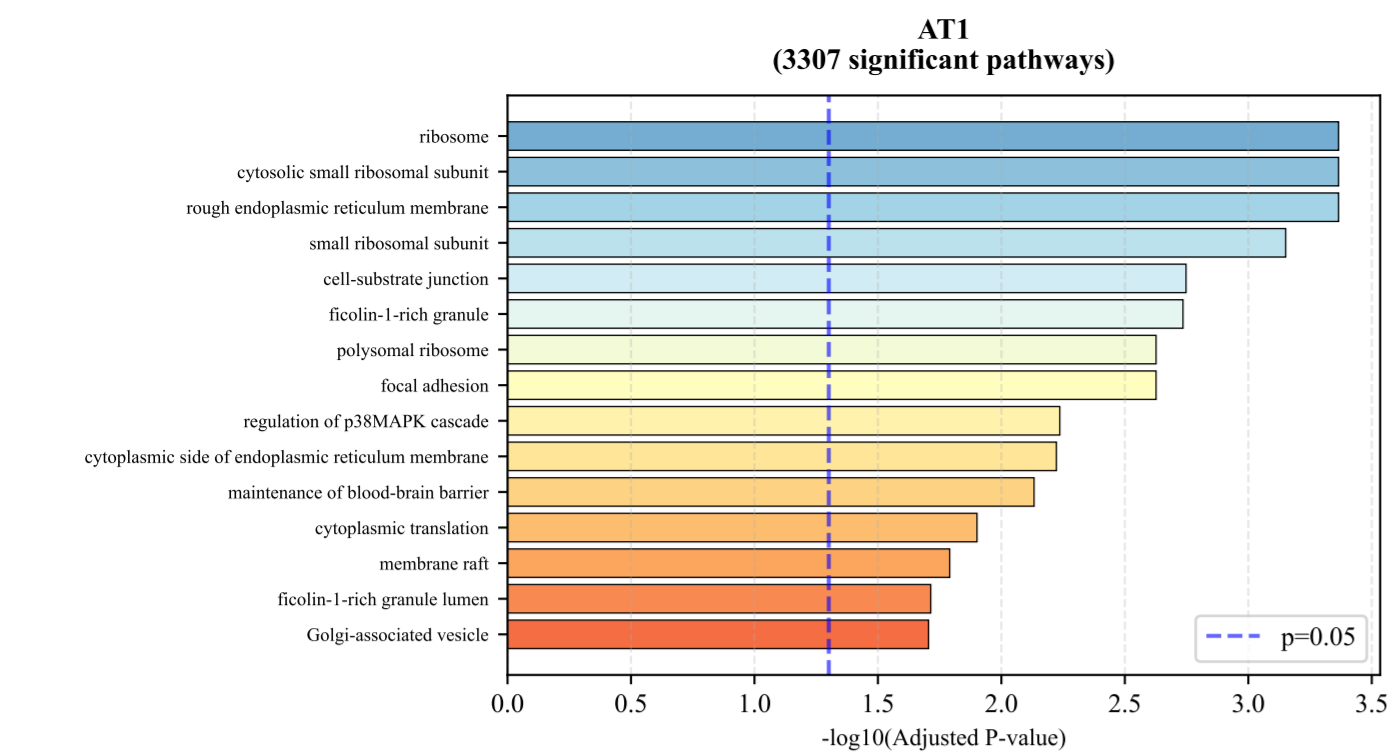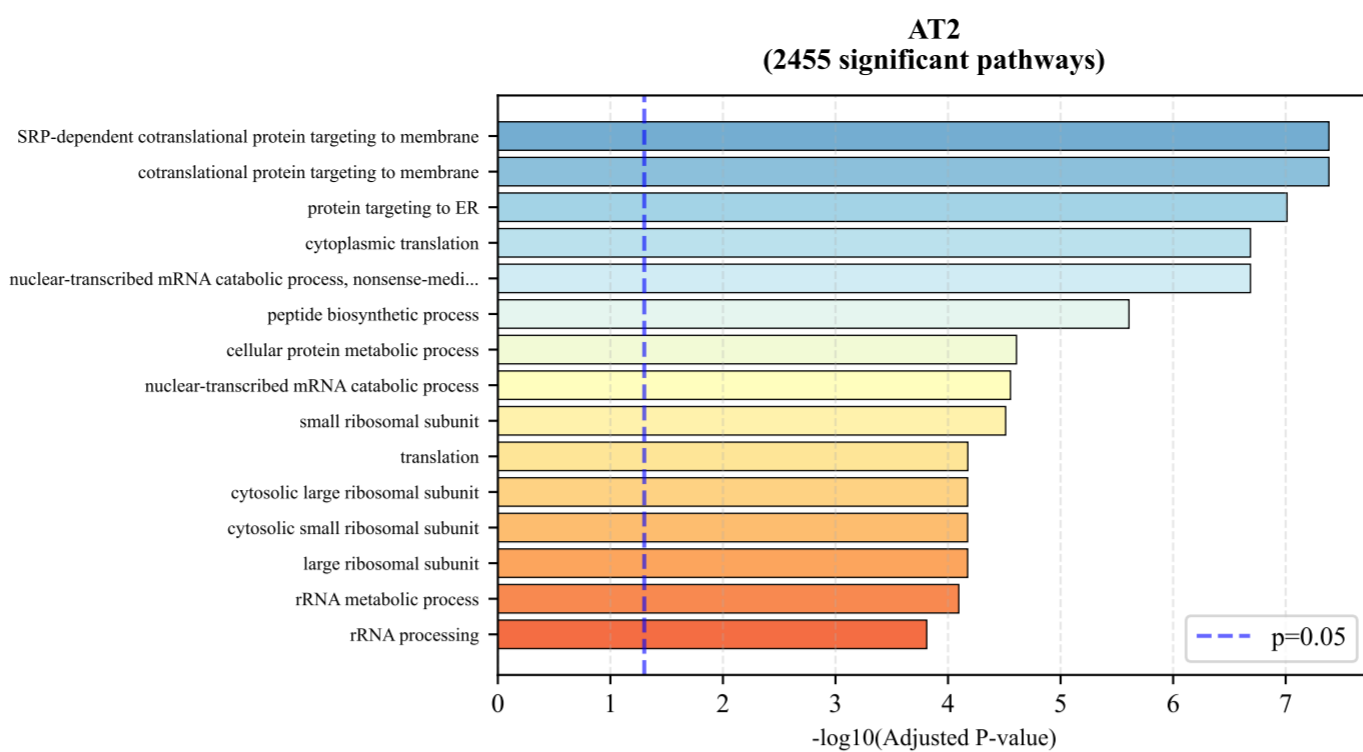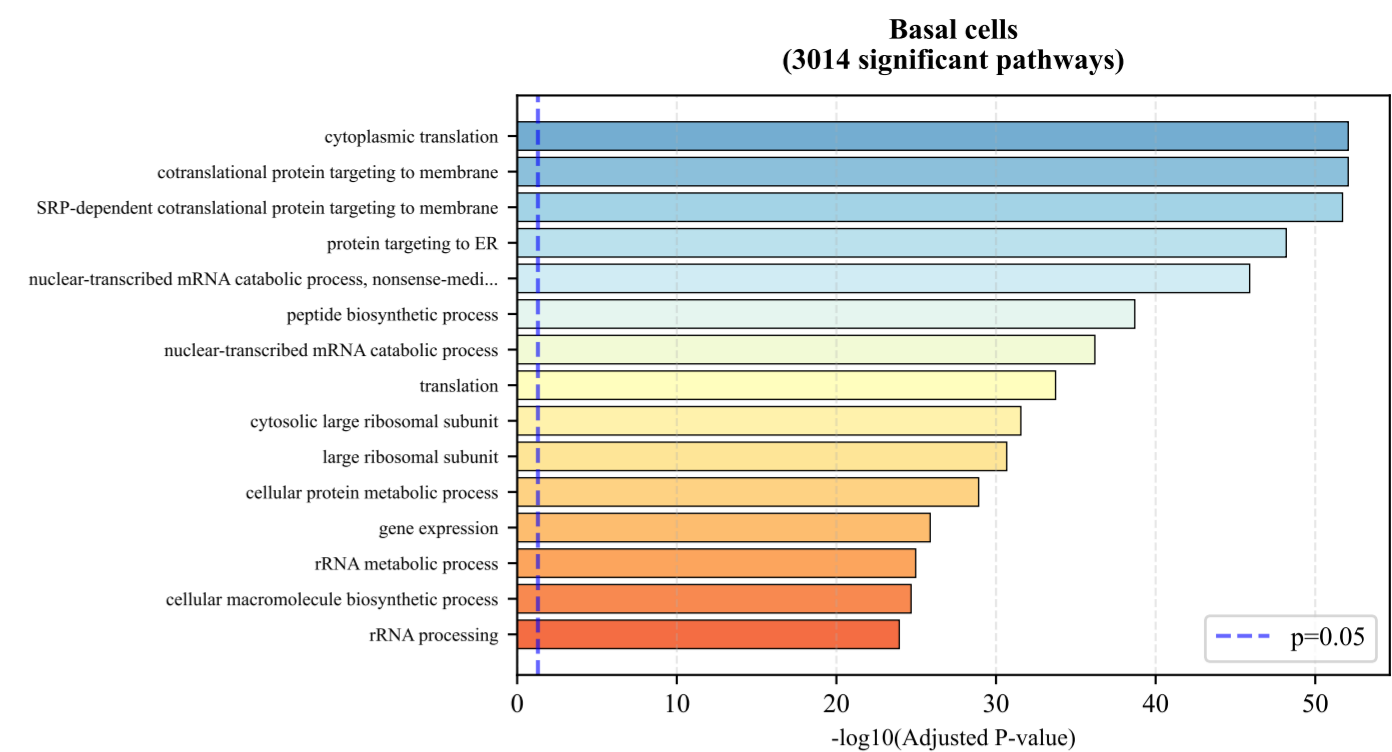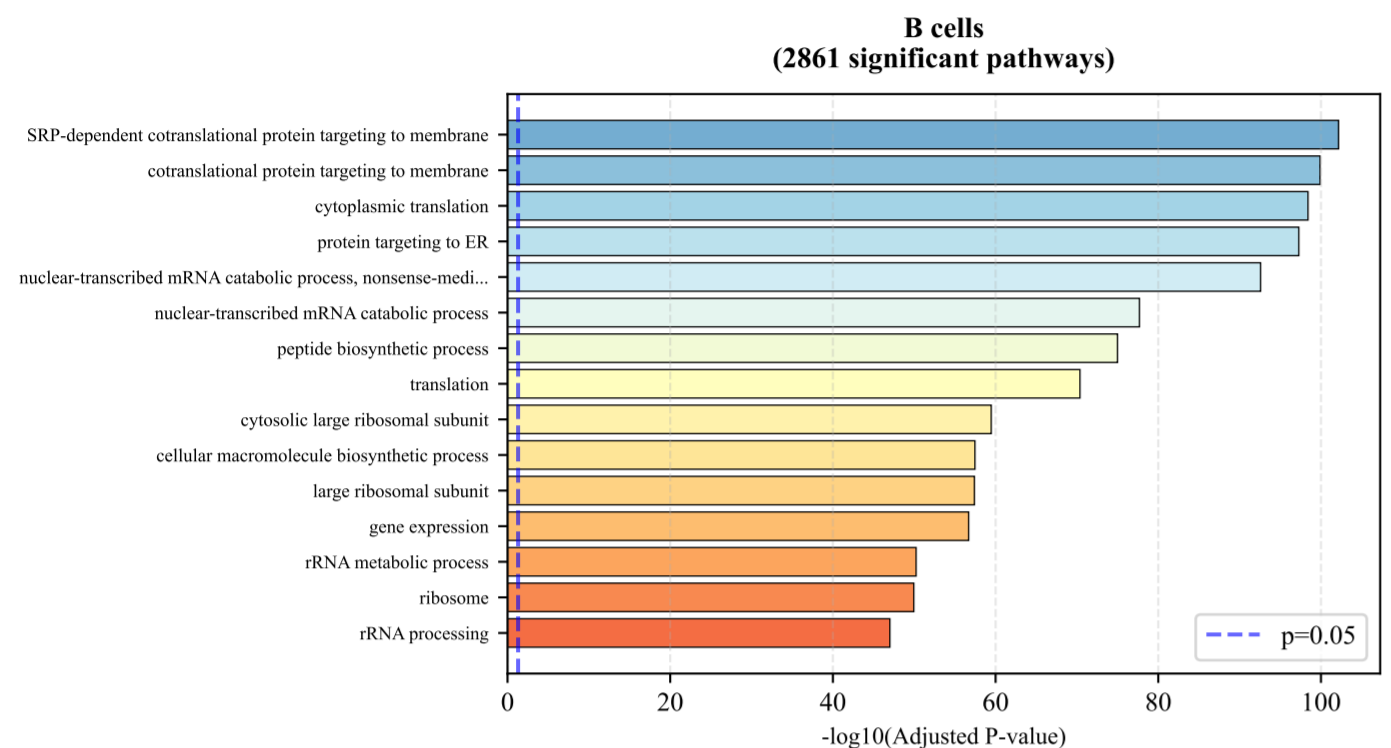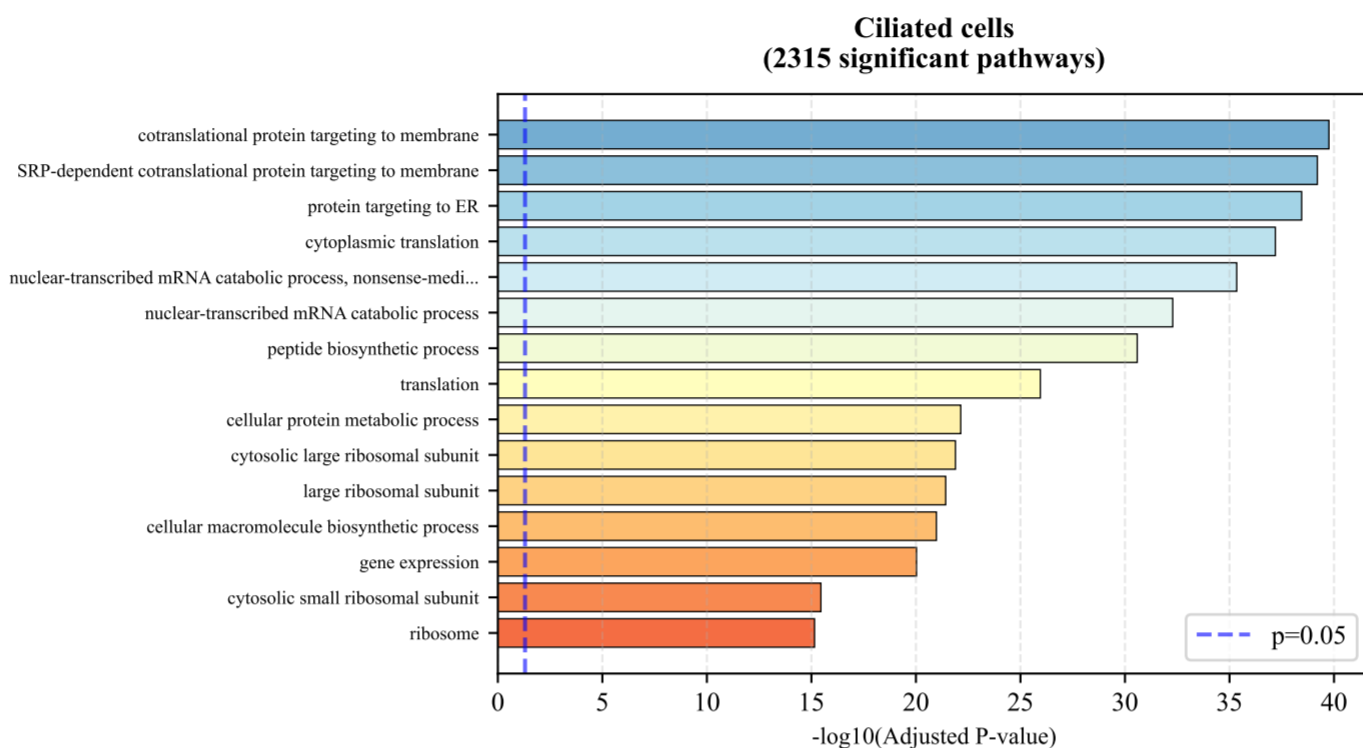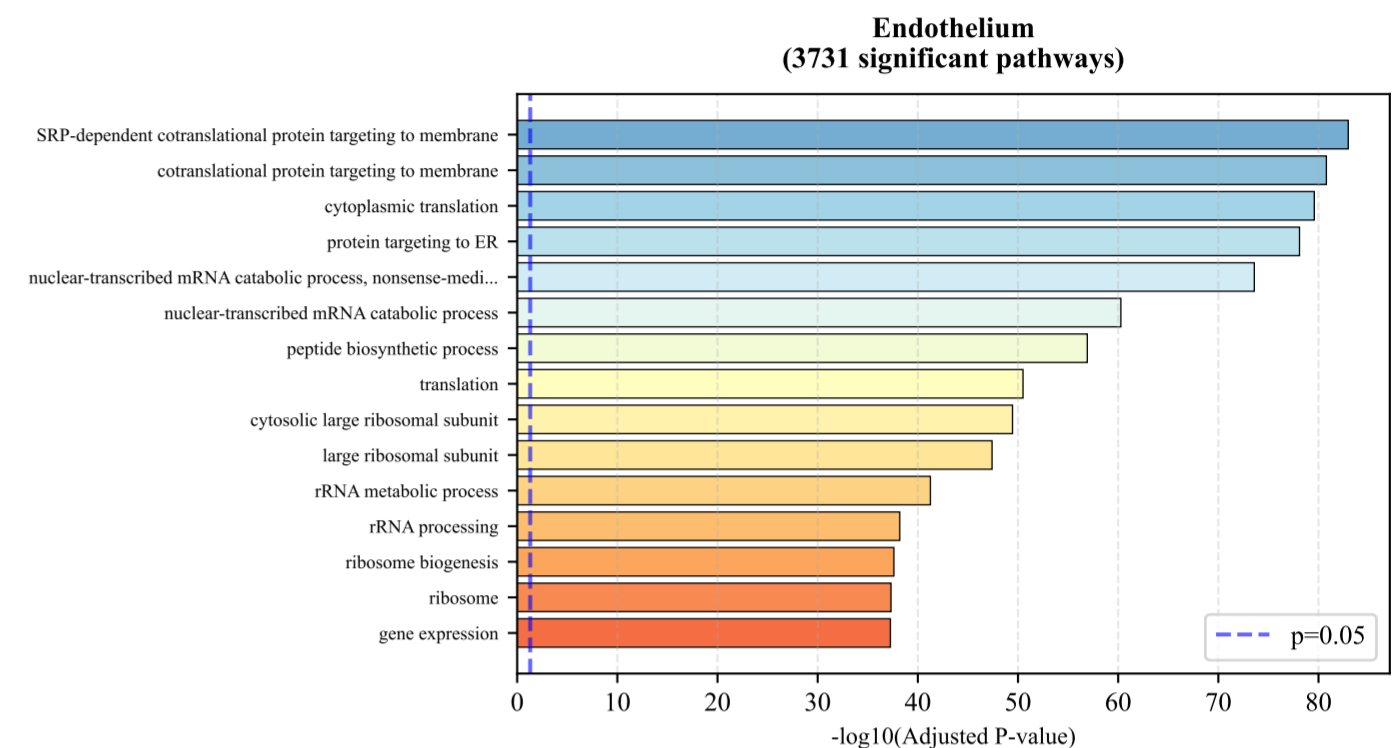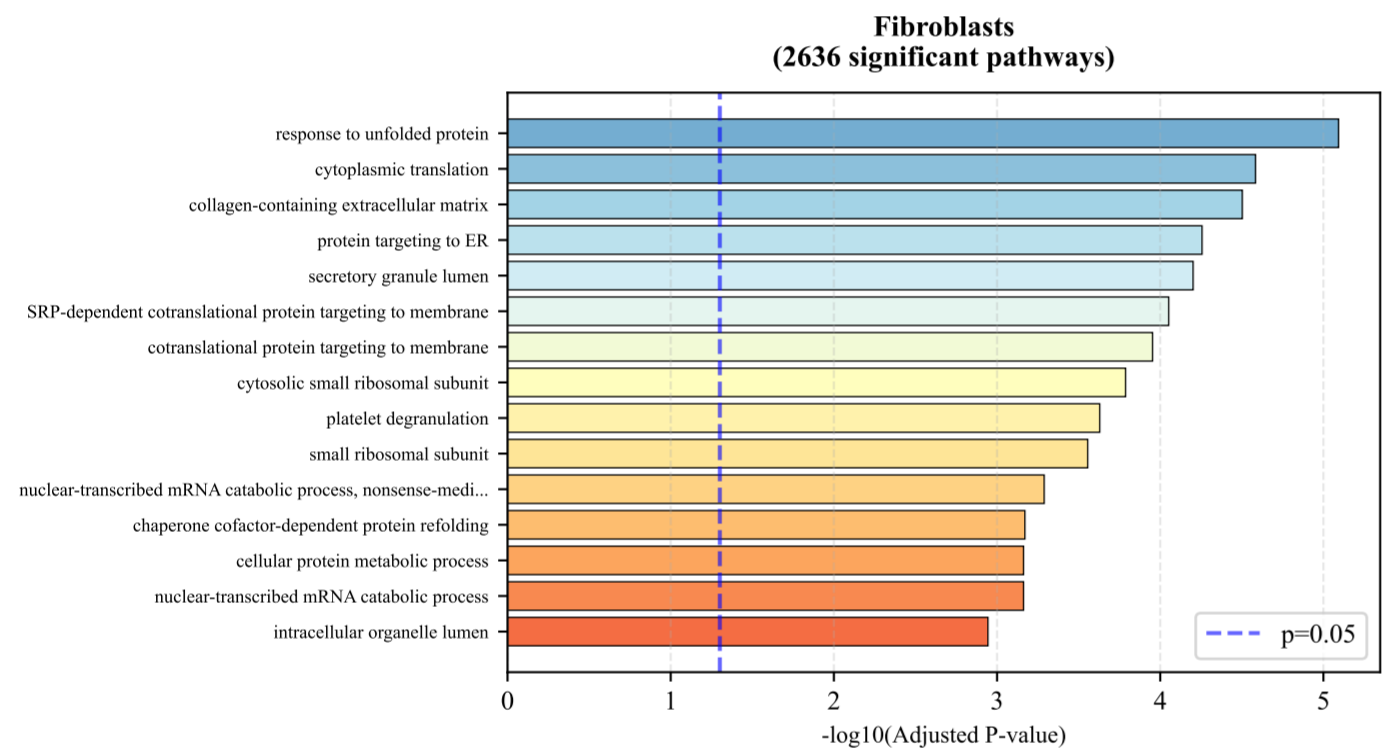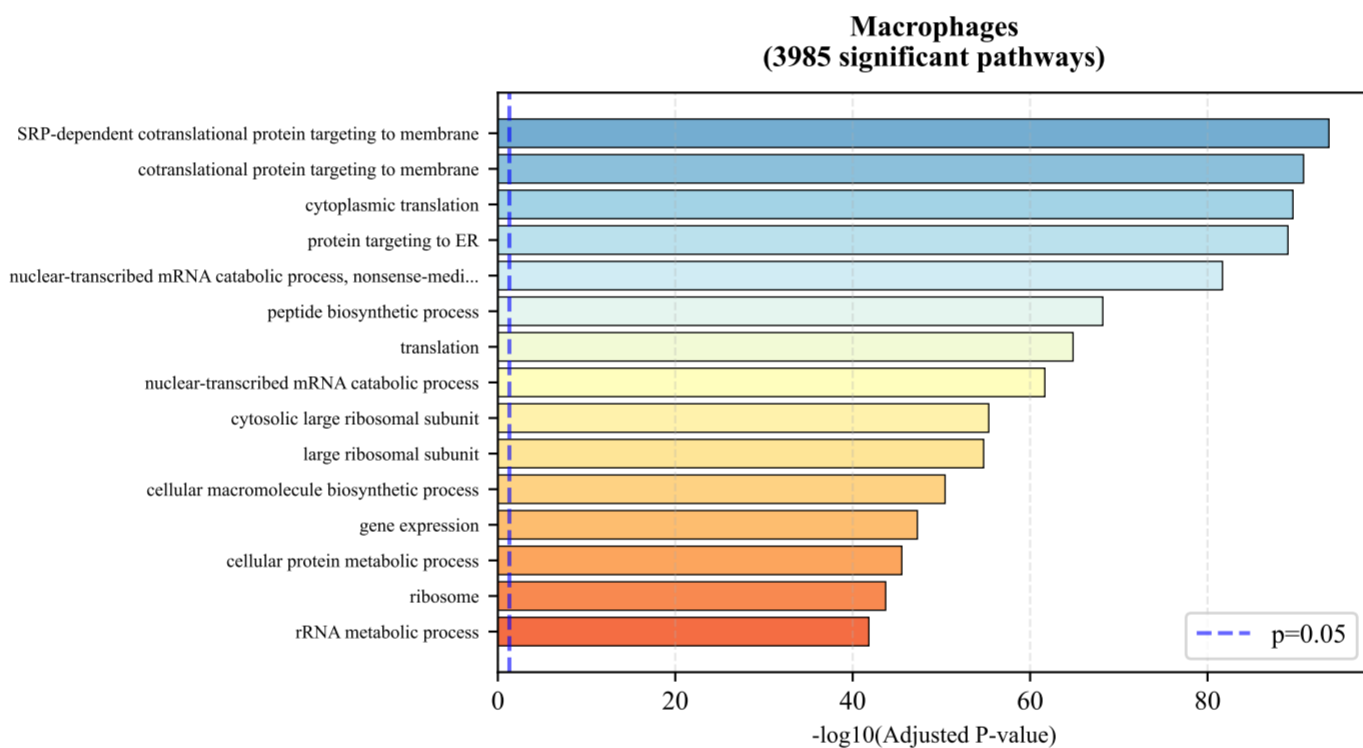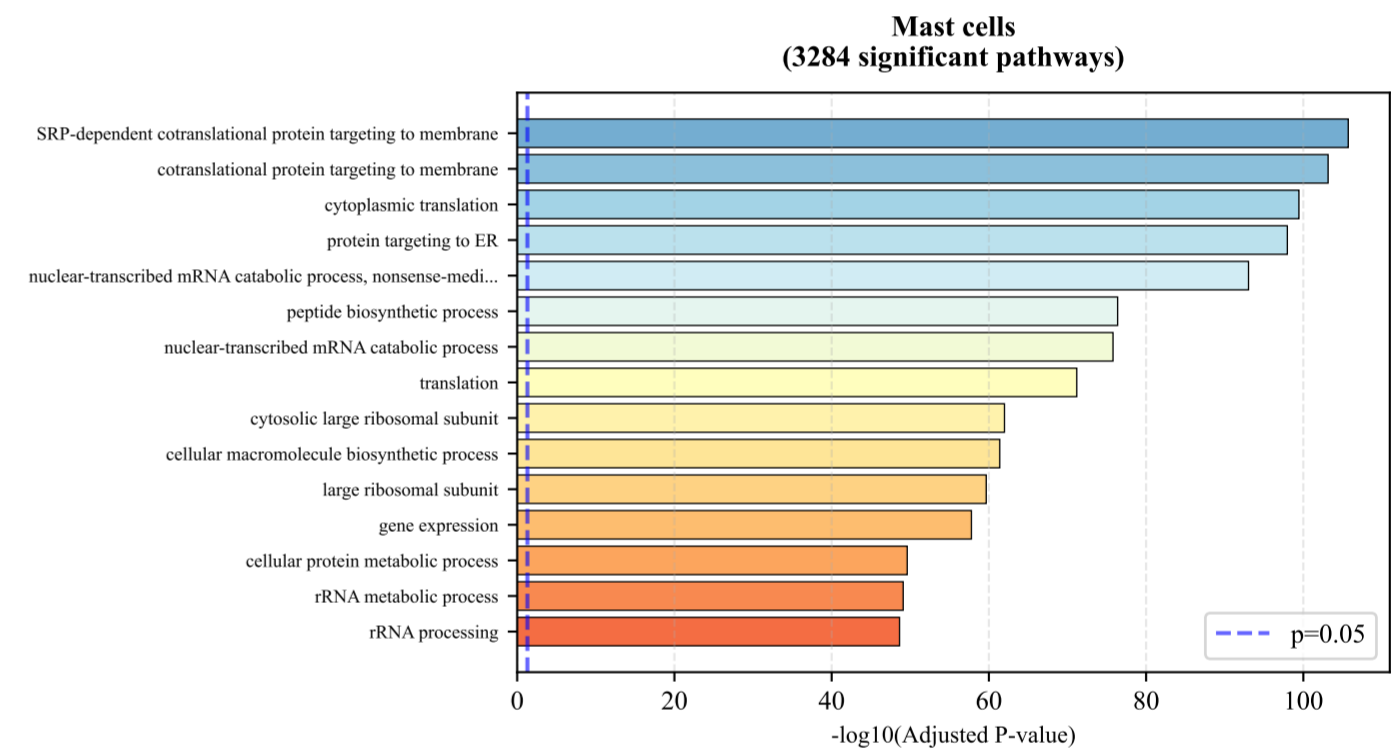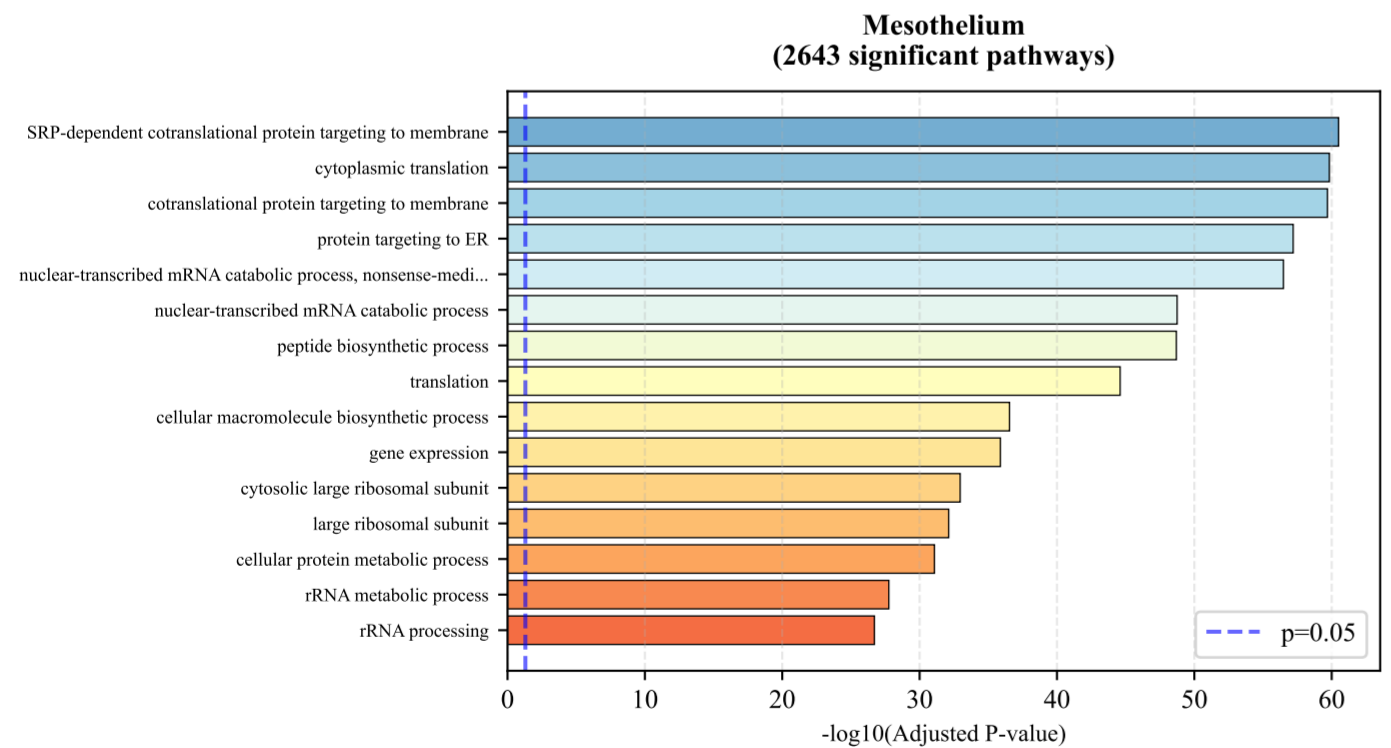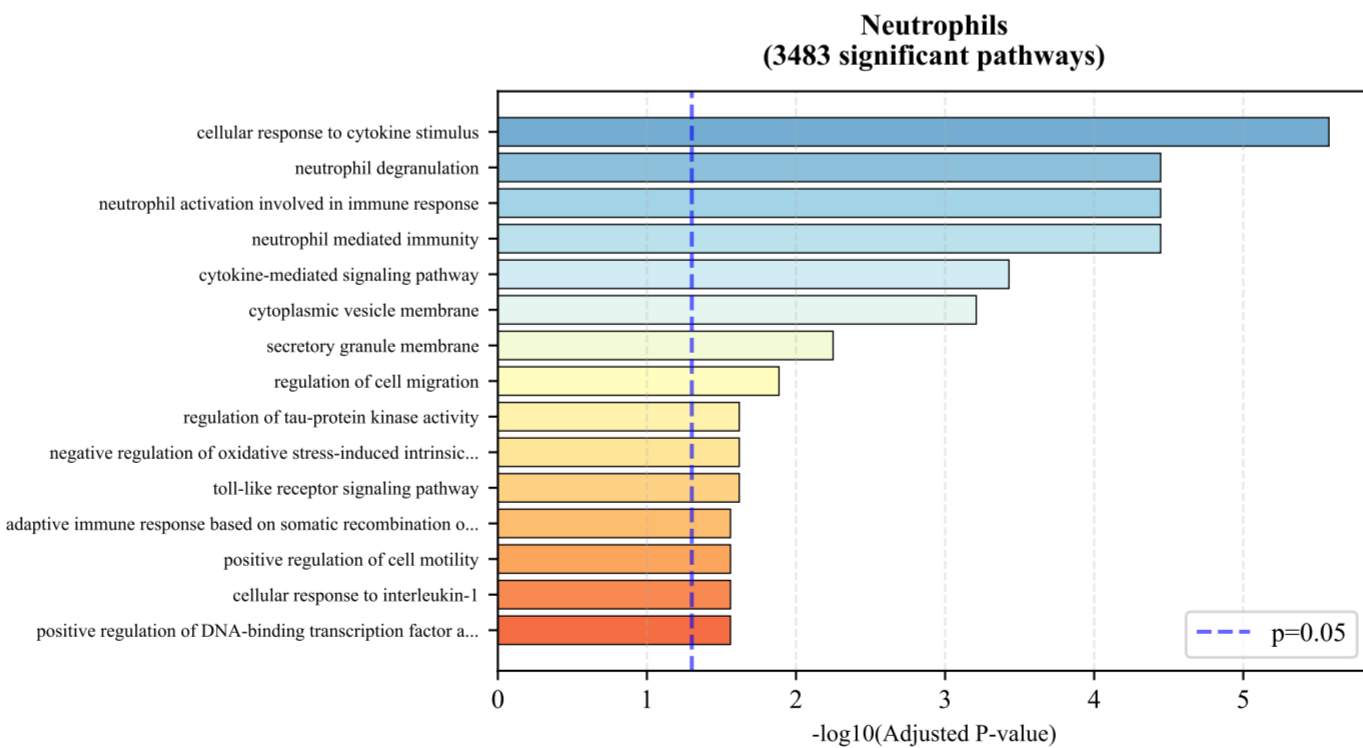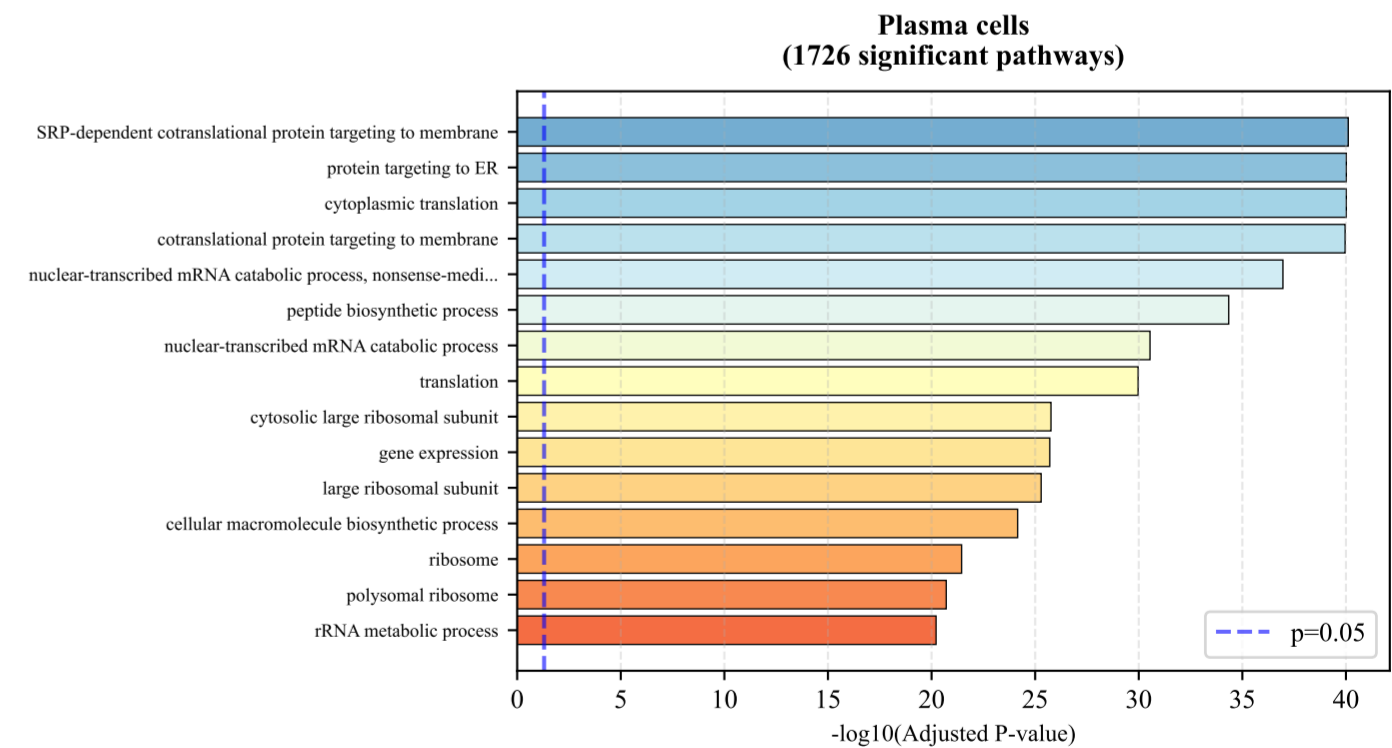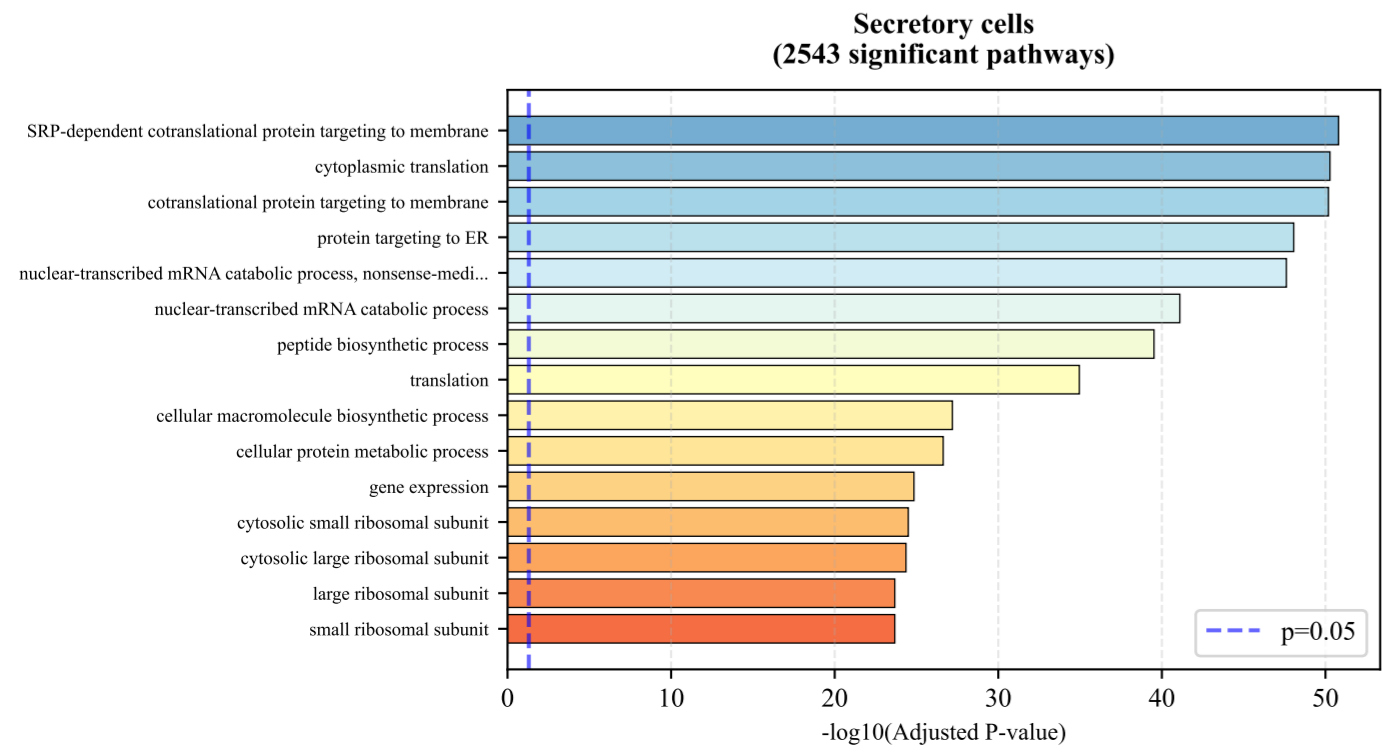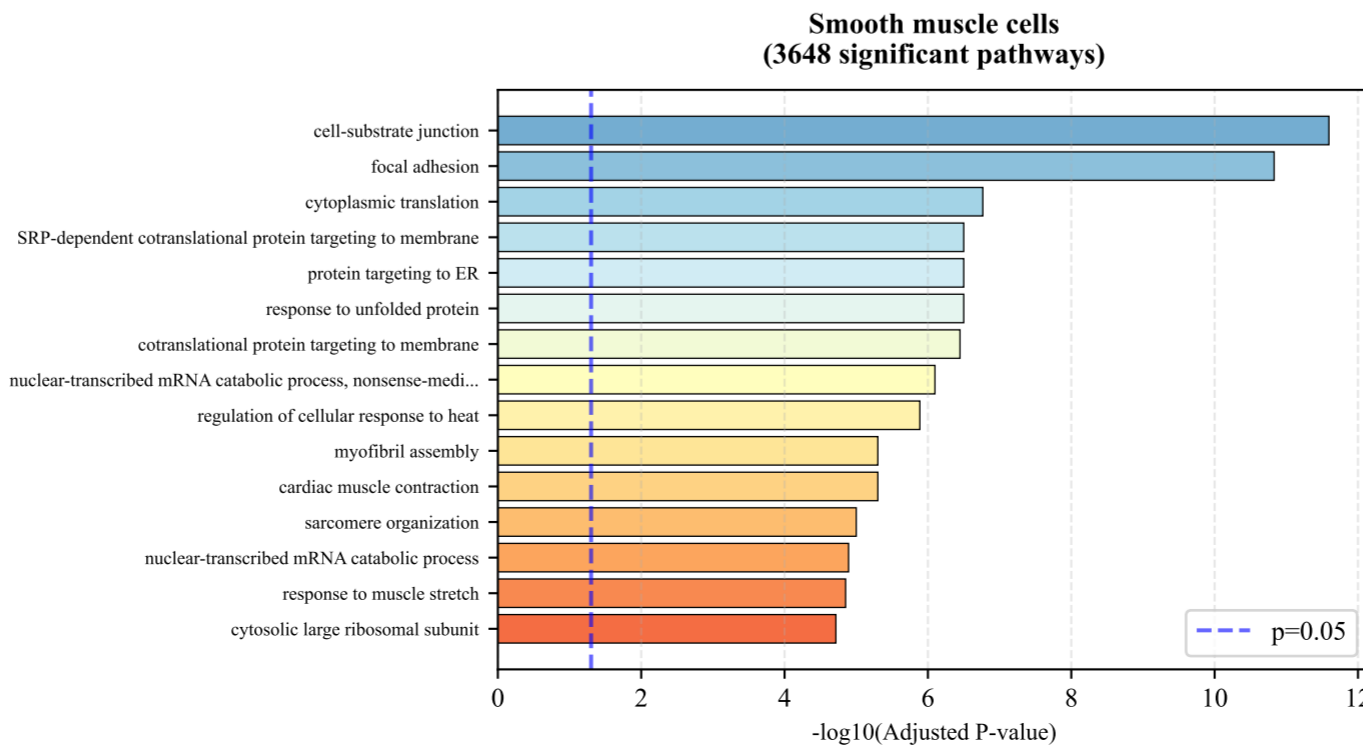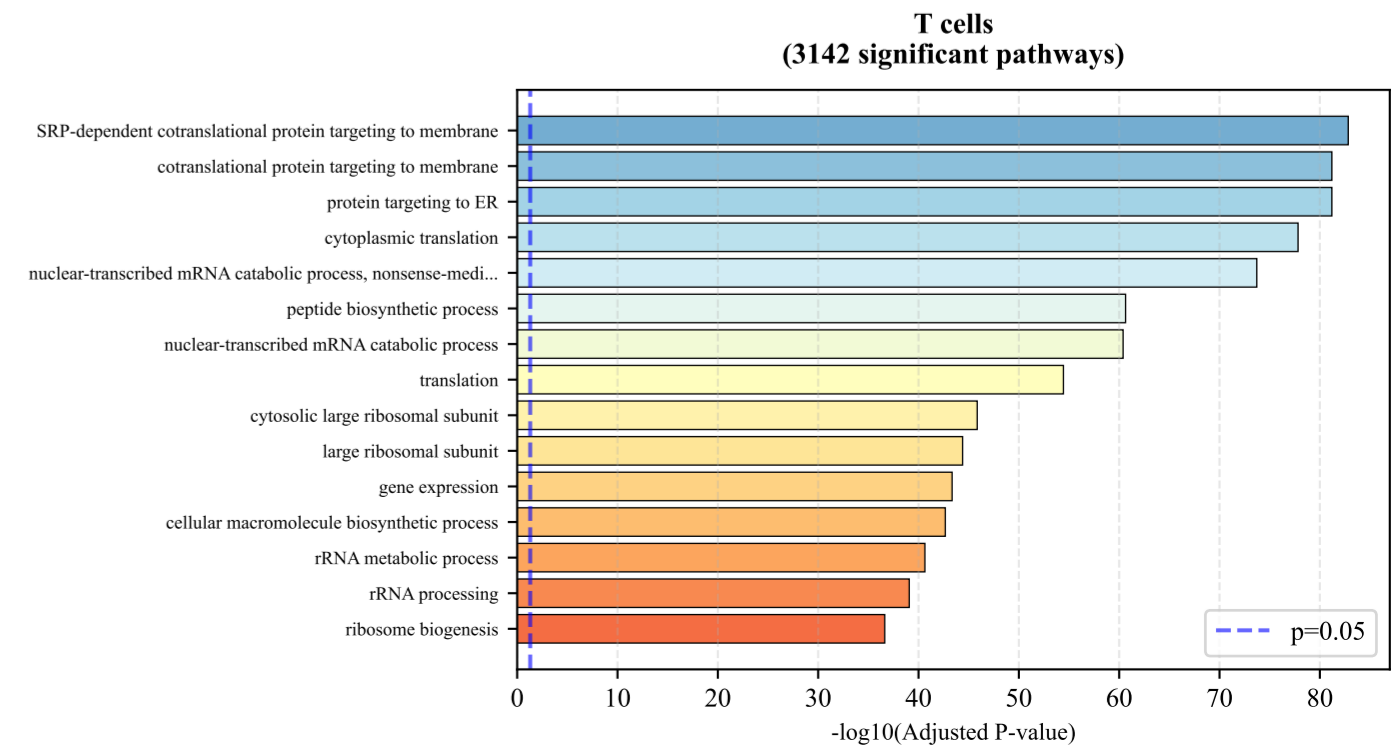

Supplement: Supplementary file 1 [file life-16-00771-s001.zip › life-4219234-supplementary/Figure S2.pdf]
